# The bZip Transcription Factor VdMRTF1 is a Negative Regulator of Melanin Biosynthesis and Virulence in Verticillium dahliae

**DOI:** 10.1128/spectrum.02581-21

**Published:** 2022-04-11

**Authors:** Meijun Lai, Zhuo Cheng, Luyao Xiao, Steven J. Klosterman, Yonglin Wang

**Affiliations:** a Beijing Key Laboratory for Forest Pest Control, College of Forestry, Beijing Forestry University, Beijing, China; b United States Department of Agriculture, Agricultural Research Service, Salinas, California, USA; Tufts University

**Keywords:** bZip transcription factor, VdMRTF1, *Verticillium dahliae*, melanized microsclerotia, virulence

## Abstract

The ascomycete fungus Verticillium dahliae infects over 400 plant species and causes serious losses of economically important crops, such as cotton and tomato, and also of woody plants, such as smoke tree, maple, and olive. Melanized long-term survival structures known as microsclerotia play crucial roles in the disease cycle of V. dahliae, enabling this soilborne fungus to survive for years in the soil in the absence of a host. Previously, we identified VdMRTF1 (microsclerotia-related transcription factor) encoding a bZip transcription factor which is downregulated during microsclerotial development in V. dahliae. In the present study, we showed that VdMRTF1 negatively controls melanin production and virulence by genetic, biological, and transcriptomic analyses. The mutant strain lacking *VdMRTF1* (Δ*VdMRTF1*) exhibited increased melanin biosynthesis and the defect also promoted microsclerotial development and sensitivity to Ca^2+^. In comparison with the wild-type strain, the Δ*VdMRTF1* strain showed a significant enhancement in virulence and displayed an increased capacity to eliminate reactive oxygen species *in planta*. Furthermore, analyses of transcriptomic profiles between the Δ*VdMRTF1* and wild-type strains indicated that VdMRTF1 regulates the differential expression of genes associated with melanin biosynthesis, tyrosine metabolism, hydrogen peroxide catabolic processes, and oxidoreductase activity in V. dahliae. Taken together, these data show that VdMRTF1 is a negative transcriptional regulator of melanin biosynthesis, microsclerotia formation, and virulence in V. dahliae.

**IMPORTANCE** Verticillium wilt is difficult to manage because the pathogen colonizes the plant xylem tissue and produces melanized microsclerotia which survive for more than 10 years in soil without a host. The molecular mechanisms underlying microsclerotia formation are of great importance to control the disease. Here, we provide evidence that a bZip transcription factor, VdMRTF1, plays important roles in melanin biosynthesis, microsclerotial development, resistance to elevated Ca^2+^ levels, and fungal virulence of V. dahliae. The findings extend and deepen our understanding of the complexities of melanin biosynthesis, microsclerotia formation, and virulence that are regulated by bZip transcription factors in V. dahliae.

## INTRODUCTION

Verticillium dahliae is a plant fungal pathogen that causes Verticillium wilt disease on more than 400 plant species, including crops, ornamentals, and forests (1, 2). Among ornamental forest trees, V. dahliae results in high mortality of smoke trees (*Cotinus coggygria* Scop.) in China (3). Verticillium dahliae produces two types of asexual propagules, conidia and long-term survival structures known as microsclerotia. An abundance of microsclerotia in soil, and their survival in adverse environmental conditions, makes *Verticillium* wilt difficult to control (1, 2). The microsclerotia germinate to produce hyphae that invade roots, proliferate, and enter the plant xylem (4).The infested plants exhibit wilt symptoms due to blockage of the water conducting xylem tissue, and may cause death of a host plant (5). Interestingly, melanin deposition is always linked with microsclerotial development and mature microsclerotia are highly melanized. Melanin is crucial for the survival of microsclerotia in response to UV irradiation and temperature fluctuations (6). Thus, elucidation of molecular bases underlying melanized microsclerotia is of great significance to shape phenotypic traits and potentially novel control strategies.

Molecular mechanisms governing microsclerotia formation and pigment production in V. dahliae have been extensively studied (1, 7, 8). Firstly, transcriptomic profiles revealed induced expression of numerous genes, especially pigment biosynthesis genes during microsclerotia formation (9, 10). Secondly, dozens of molecular genetics analyses have pinpointed genes involved in microsclerotia formation, many of which either regulate transcription or signal transduction (7). For instance, VMK1 encoding a mitogen-activated protein (MAP) kinase is important for multiple cellular process such as conidiation and microsclerotia formation in V. dahliae (11). Apart from VdHog1, VdPbs2, VdSho1, and VdSte11 (12–15), many transcription factors, such as Vdste12 (16), VdMsn2 (17), VdCrm1 (6), and VdYap1 (18), have been identified to play important roles in microsclerotia formation.

The process of melanin biosynthesis involves oxidative polymerization of phenolic and indole compounds and, in some fungi, is associated with virulence and/or survival under extreme environmental conditions (19–21). There are several types of melanin, including 1,8-dihydroxynaphthalene melanin (DHN-melanin), hydroxyphenylalanine melanin (DOPA-melanin), and pyomelanin (19, 22). The major type of melanin produced among fungi, such as Botrytis cinerea and V. dahliae, is DHN-melanin, which is synthesized from acetyl coenzyme A via the polyketide pathway (19, 23, 24). Some fungi, such as Aspergillus nidulans, *Penicillium marneffei*, and Cryptococcus neoformans can produce DOPA-melanin (25–27). Another type of melanin, named pyomelanin, is water-soluble, and it may be produced via the tyrosine degradation pathway(28, 29). Fungi such as and Aspergillus fumigatus can produce pyomelanin. (28, 29). Melanin acts as a virulence factor in some fungi such as Magnaporthe grisea, *Sporothrix schenckii* and C. neoformans (19, 30, 31). In V. dahliae strain V592, deletion of the polyketide synthase *VdPKS1*, which is required for melanin biosynthesis, resulted in reduced virulence (32). However, deletion of either *VdPKS1* and *VdCmr1*, which are required for melanin production in strain VdLs.17 had no effect on pathogenesis in tobacco or lettuce (6). Therefore, the linkage between DHN-melanin production in V. dahliae and virulence requires further research.

In one of our previous studies, a bZip transcription factor (VDAG_09790, VdMRTF1) was downregulated in microsclerotia-producing, pigmented colonies of V. dahliae (33). This current study aimed to reveal the functions of VdMRTF1 in V. dahliae. Deletion of *VdMRTF1* led to induced expression of melanin biosynthesis genes and resulted in an increase in virulence. Furthermore, RNA-seq analyses indicated that VdMRTF1 is involved in the regulation of tyrosine metabolism and in eliminating reactive oxygen species. These results demonstrated that the bZip transcription factor VdMRTF1 is a negative transcriptional regulator for melanin biosynthesis, microsclerotia formation, and pathogenicity in V. dahliae.

## RESULTS

### Loss of *VdMRTF1* increases melanin biosynthesis.

Results from our previous transcriptomic data indicated that VDAG_09790, encoding a bZip transcription factor, was downregulated during microsclerotia production (33). Here, VDAG_09790 was designated as microsclerotia-related transcription factor, VdMRTF1. Amino acid alignments and phylogenetic analyses revealed a high degree of sequence similarity of VdMRTF1 compared with its homologs in other fungi (Fig. S1), demonstrating that MRTF1 is relatively conserved among these fungal species. To functionally investigate the roles of VdMRTF1, deletion mutants (Δ*VdMRTF1*) and complemented strain (Δ*VdMRTF1*-C) were obtained. Gene replacement by homologous recombination in the Δ*VdMRTF1* strain and complementation in the Δ*VdMRTF1*-C strain were verified by multiple PCR analyses and DNA blots (Fig. S2).

**FIG 1:**
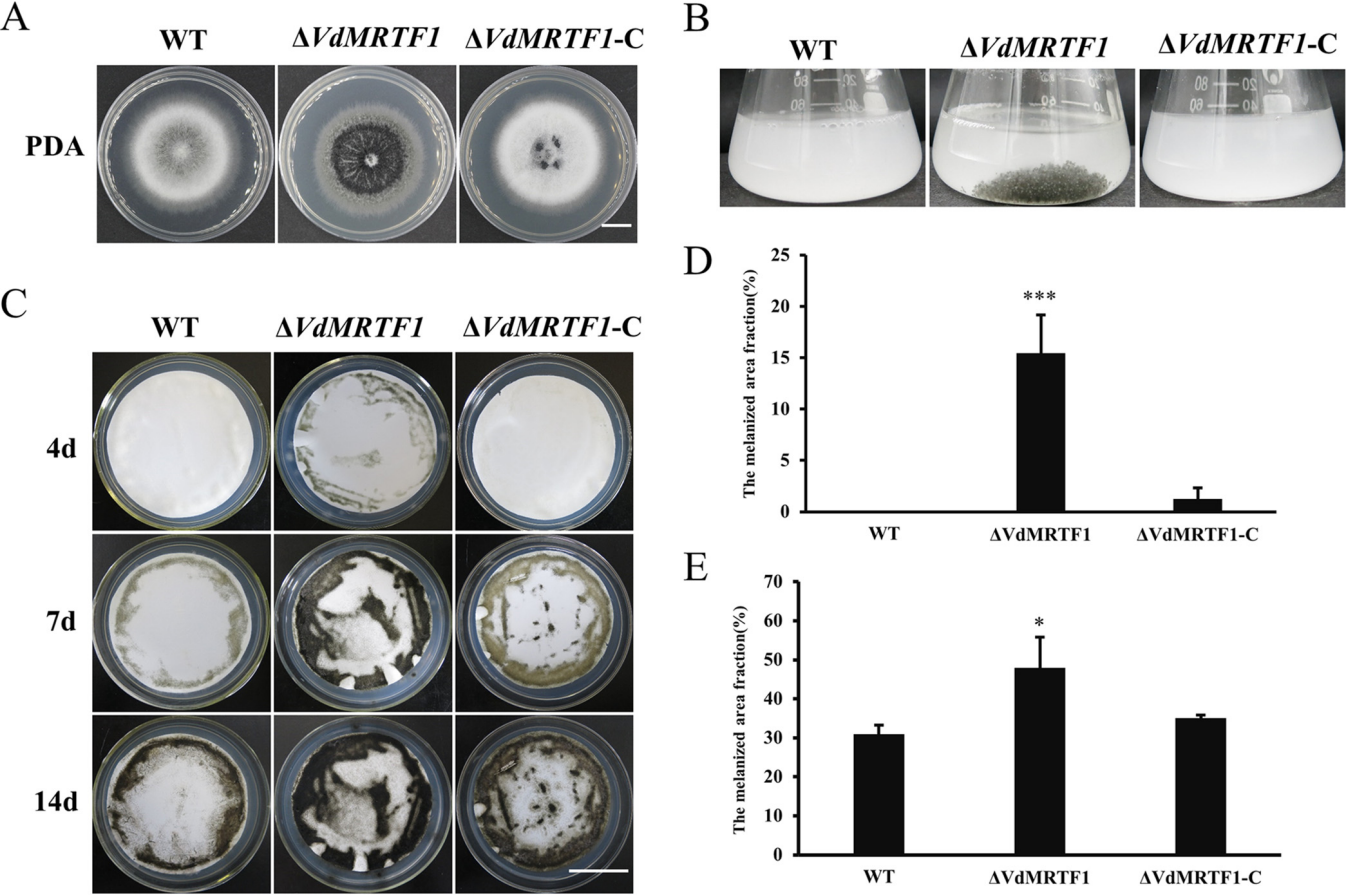
*VdMRTF1* negatively regulates melanin biosynthesis in Verticillium dahliae. (A) Colonies of the wild-type strain (VdLs.17), the Δ*VdMRTF1* strain, and the complemented strain (Δ*VdMRTF1*/*VdMRTF1*) were grown on PDA for 10 days at 25°C. Bar, 1 cm. (B) Conidial suspensions (10^6^/mL) of the indicated strains of V. dahliae were shaken for 10 days at 25°C in liquid BM. (C) Conidia (10^6^/mL) of each strain were sprayed on the cellulose membrane overlaid onto BM plates and cultured at 25°C. Photographs were taken after 4, 7, and 14 days. Bar, 3 cm. (D) and (E) Melanized fractions of the colonies were determined using ImageJ at 4 and 14 dpi. Error bars represent the standard deviations based on three independent replicates. Asterisks indicate significant differences (***P < 0.001, *P < 0.5).

Phenotypic observations of the wild-type VdLs.17, ΔVdMRTF1, and ΔVdMRTF1-C strains grown on potato dextrose agar medium (PDA) for 10 days indicated that the Δ*VdMRTF1* strain accumulated increased amounts of melanin in the center of the colonies, while the wild-type strain and the Δ*VdMRTF1*-C strain accumulated less melanin than the ΔVdMRTF1 strain (Fig. 1A). To further examine the specific role of VdMRTF1 in melanin synthesis, the conidial suspensions of the wild-type, Δ*VdMRTF1* mutant, and complemented strain were added into liquid basal medium (BM) medium, a medium conducive to microsclerotia formation and pigment production in V. dahliae (10). The Δ*VdMRTF1* strain formed melanized microsclerotia while the wild-type and the Δ*VdMRTF1*-C strain did not form melanized microsclerotia on BM (Fig. 1B). Additionally, the conidial suspensions of all strains were uniformly sprayed on a cellulose membrane overlaid onto BM plates to observe melanin synthesis. The result implied that melanin appeared in the Δ*VdMRTF1* strain at 4 days postinoculation (dpi), but the wild-type and the Δ*VdMRTF1*-C strain rarely formed melanin (Fig. 1C and D). These observations were continued for up to 14 dpi; both the wild-type and the Δ*VdMRTF1* strains formed more and more melanin during this extended period (Fig. 1C). However, the melanized fraction of the Δ*VdMRTF1* strain (48% at 14 dpi) was larger than the wild-type strain (31% at 14 dpi) on BM plates, the Δ*VdMRTF1*-C strain showed similar (35% at 14 dpi) to the wild-type strain (Fig. 1C and E). In summary, these results suggest that VdMRTF1 is important but not required for melanin synthesis in V. dahliae.

### *VdMRTF1* is involved in microsclerotia formation.

**FIG 2:**
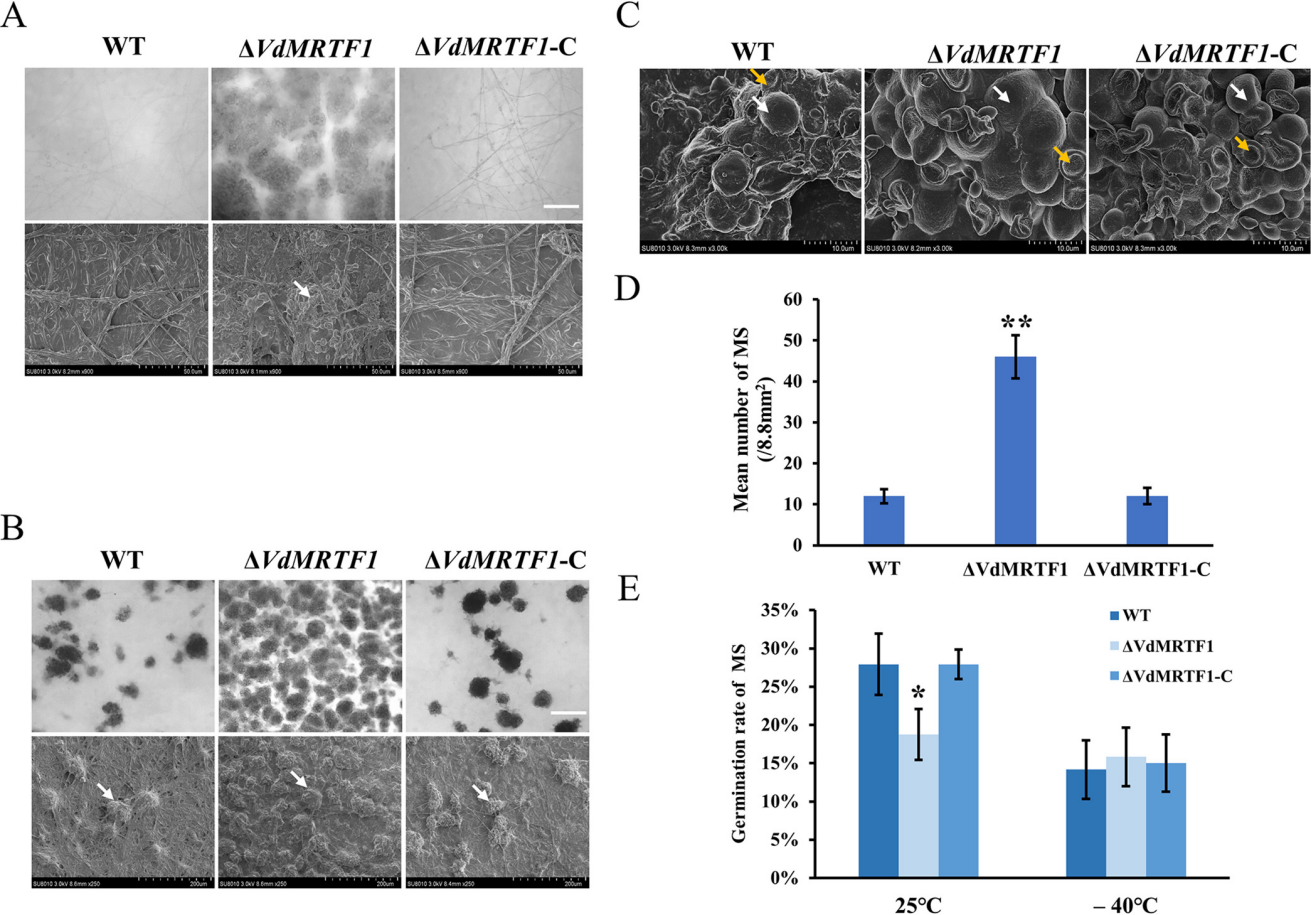
Microsclerotia formation, germination, and morphology of wild-type, Δ*VdMRTF1*, and Δ*VdMRTF1-*complemented strains of Verticillium dahliae. (A) Microsclerotium formation of the wild-type, Δ*VdMRTF1*, and complemented (*ΔVdMRTF1-*C) strains were captured by biological microscope (above) and scanning electron microscopy(below) on BM at 25°C for 4 days. White arrow points to a microsclerotium. Bar = 100 μm. (B) Microsclerotium formation of the wild-type, Δ*VdMRTF1*, and ΔVdMRTF1-C strains were captured by biological microscope (above) and scanning electron microscopy(below) on BM at 25°C for 7 days. White arrow points to a microsclerotium. Bar = 100 μm. (C) The morphology of 14-day-old microsclerotia incubation on BM by scanning electron microscopy. White arrow points to plump microsclerotia, and the orange arrow points to widened microsclerotia. (D) The bar chart showed the mean number of microsclerotium (MS) of all strains on BM at 25°C for 7 days. Error bars represent the standard deviations of three replicates. Asterisks indicate significant differences (**P < 0.01). (E) The bar chart showed the germination rate of microsclerotium (MS) of the wild-type, Δ*VdMRTF1*, and ΔVdMRTF1-C strains in different temperature. Error bars represent the standard deviations based on three independent replicates. Asterisks indicate significant differences (*P < 0.05).

To further explore the function of VdMRTF1 in the regulation of microsclerotia formation, we examined the samples in Fig. 1C by light microscopy and scanning electron microscopy (SEM). Microscopic observations showed that numerous melanized microsclerotia appeared in clusters in the Δ*VdMRTF1* strain at 4 dpi, while melanized microsclerotia were almost invisible in the wild-type strain and the complemented strain (Fig. 2A). These observations were carried out up to 7 days; microscopic examination showed that the Δ*VdMRTF1* strain produced much more melanized microsclerotia than the wild-type and the Δ*VdMRTF1*-C strains (Fig. 2B). Further quantification by ImageJ demonstrated that the Δ*VdMRTF1* strain produced 5 times as much melanized microsclerotia as the wild-type strain and the Δ*VdMRTF1*-C strain (Fig. 2D). According to our previous research, deletion of some transcription factors can influence the stability of the microsclerotia, and there are two types of cells in microsclerotia: one type was rounded and plump, and the other was widened (11). To investigate whether the VdMRTF1 plays a role in morphological development of microsclerotia, we observed the morphology of 14-day-old microsclerotia on cellulose membranes by SEM. Both the plump and widened types of microsclerotia were observed in the wild-type, the Δ*VdMRTF1*, and the Δ*VdMRTF1*-C strains (Fig. 2C). In addition, we tested the germination rate of the microsclerotia of all strains, revealing that the germination rate of microsclerotia in the Δ*VdMRTF1* strain is 18.8%, which was lower than that in the wild-type strain (27.9%) and the Δ*VdMRTF1*-C strain (27.9%) at 25°C. In contrast, there was no significance difference between all strains when microsclerotium were exposed in –40°C for 2 days (Fig. 2E). These results demonstrated that VdMRTF1 regulates the formation and germination rate of microsclerotia, but not their morphological development.

### VdMRTF1 is involved in resistance to elevated Ca^2+^ in V. dahliae.

**FIG 3:**
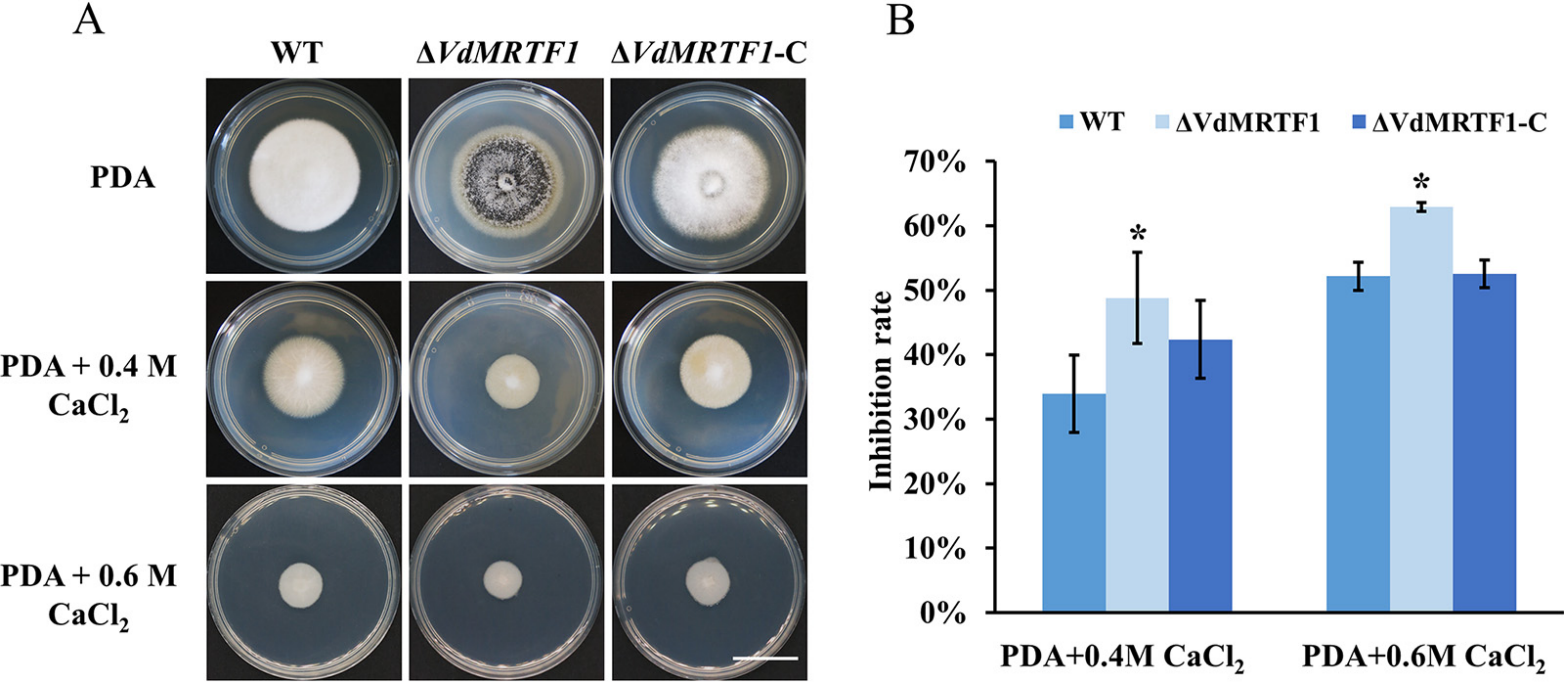
VdMRTF1 is required for resistance to Ca^2+^ in Verticillium dahliae. (A) The wild-type, Δ*VdMRTF1*, and Δ*VdMRTF1*-C strains were cultured in PDA, PDA with 0.4M CaCl_2_, and 0.6M CaCl_2_ at 25°C for 10 days. Photographs were taken at 10 dpi. Bar, 2 cm. (B) The bar chart showed the inhibition rate of the wild-type, Δ*VdMRTF1*, and Δ*VdMRTF1*-C strains under different concentrations of CaCl_2_. Error bars represent the standard deviations based on three independent replicates. Asterisks indicate significant differences (*P < 0.05).

To determine whether VdMRTF1 plays a role in tolerance to elevated Ca^2+^ levels, we cultured the wild-type VdLs.17, Δ*VdMRTF1*, and Δ*VdMRTF1*-C strains on PDA containing 0.4M and 0.6M Ca^2+^. The colonies of the Δ*VdMRTF1* strain were notably reduced in size compared with the wild-type VdLs.17 strain on PDA containing 0.4M and 0.6M Ca^2+^ (Fig. 3A). The inhibition rate of the Δ*VdMRTF1* strain cultured in PDA containing 0.4M Ca^2+^ (48.8% for the Δ*VdMRTF1* strain; 34.0% for the wild-type strain) or 0.6M Ca^2+^ (62.9% for the Δ*VdMRTF1* strain; 52.2% for the wild-type strain) was significantly higher than that of the wild-type strain. The phenotype of the Δ*VdMRTF1*-C strain was similar to the wild-type VdLs.17 strain (Fig. 3B). These results indicated that VdMRTF1 plays a role in resistance to Ca^2+^ in V. dahliae.

### VdMRTF1 negatively regulates virulence of V. dahliae.

**FIG 4:**
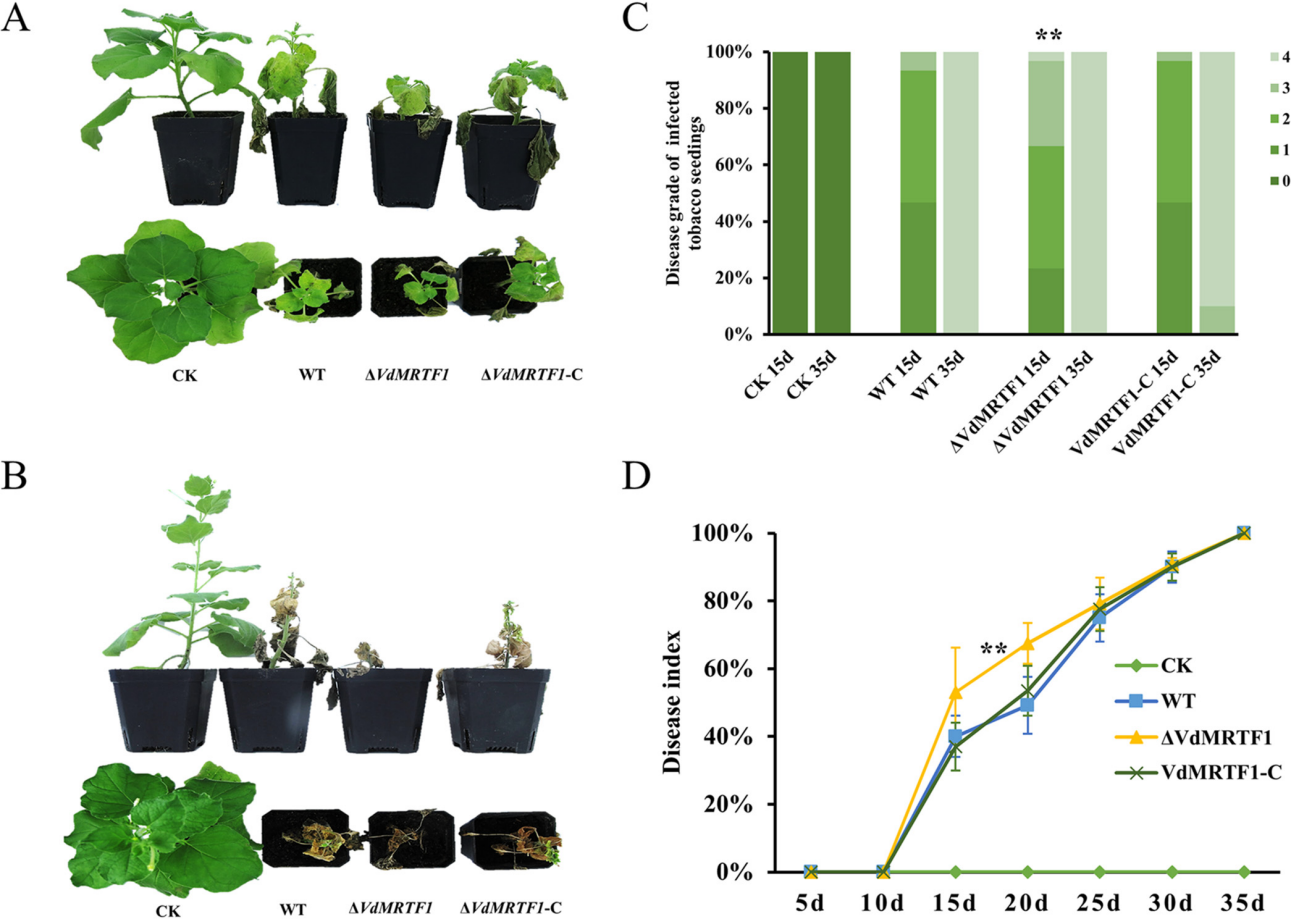
Deletion of *VdMRTF1* in Verticillium dahliae increases its virulence on tobacco seedlings. (A) 1-month-old tobacco seedlings (Nicotiana benthamiana) were inoculated and incubated for 10 min with a 10^6^-conidium/mL suspension of the wild-type VdLs.17, Δ*VdMRTF1*, and Δ*VdMRTF1*-C strains. Controls (CK) were mock-inoculated with distilled water. Disease symptoms of tobacco seedlings were photographed at 15 dpi. (B) Disease symptoms of tobacco seedlings were photographed at 35 dpi. (C) Disease symptoms of tobacco seedlings were scored visually on a scale from 0 to 4 at 15 dpi and 35 dpi (0 represents no symptoms, 1 represents one third of leaves with chlorosis or wilting, 2 represents one half leaves with chlorosis or wilting, 3 represents two thirds of leaves with chlorosis or wilting, and 4 represents more than 85% leaves with chlorosis or wilting). The results were based on three independent replicates. Asterisks indicate significant differences (**P < 0.01). (D) Line chart showing the disease index of tobacco seedlings inoculated with wild-type VdLs.17, Δ*VdMRTF1*, and Δ*VdMRTF1*-C strains. The results were based on three independent replicates. Error bars represent the standard deviations based on three independent replicates. Asterisks indicate significant differences (**P < 0.01).

To determine whether VdMRTF1 contributes to virulence, we carried out virulence assays using tobacco. Tobacco seedlings were inoculated with spore suspensions from the wild-type VdLs.17, Δ*VdMRTF1*, and Δ*VdMRTF1*-C strains. At 15 dpi, the tobacco seedlings inoculated with the Δ*VdMRTF1* spores displayed more serious disease symptoms than either the wild-type or Δ*VdMRTF1*-C strains. The symptom level of the diseased tobacco seedlings inoculated with the Δ*VdMRTF1* strain reached severity level 4, whereas the symptom level of the most seriously diseased tobacco seedlings inoculated with the wild-type and Δ*VdMRTF1*-C spores was 3 (Fig. 4A and C). At 35 dpi, tobacco seedlings infected with the wild-type, the Δ*VdMRTF1* and the Δ*VdMRTF1*-C strains showed obvious wilt symptoms, and almost all tobacco seedlings displayed a symptom level of 4 (Fig. 4B and C). The disease index of tobacco seedlings inoculated with the Δ*VdMRTF1* strain was higher than that observed following inoculation with the wild-type strain from 15 dpi (53.0% for the Δ*VdMRTF1* strain; 40.0% for the wild-type strain) to 20 dpi (67.5% for the Δ*VdMRTF1* strain; 47.2% for the wild-type strain). The disease index following inoculation of the Δ*VdMRTF1*-C strain was similar to the wild-type strain (Fig. 4D). Together, these data demonstrated that VdMRTF1 negatively regulates virulence of V. dahliae.

### VdMRTF1 is involved in the elimination of endogenous and exogenous reactive oxygen species.

**FIG 5:**
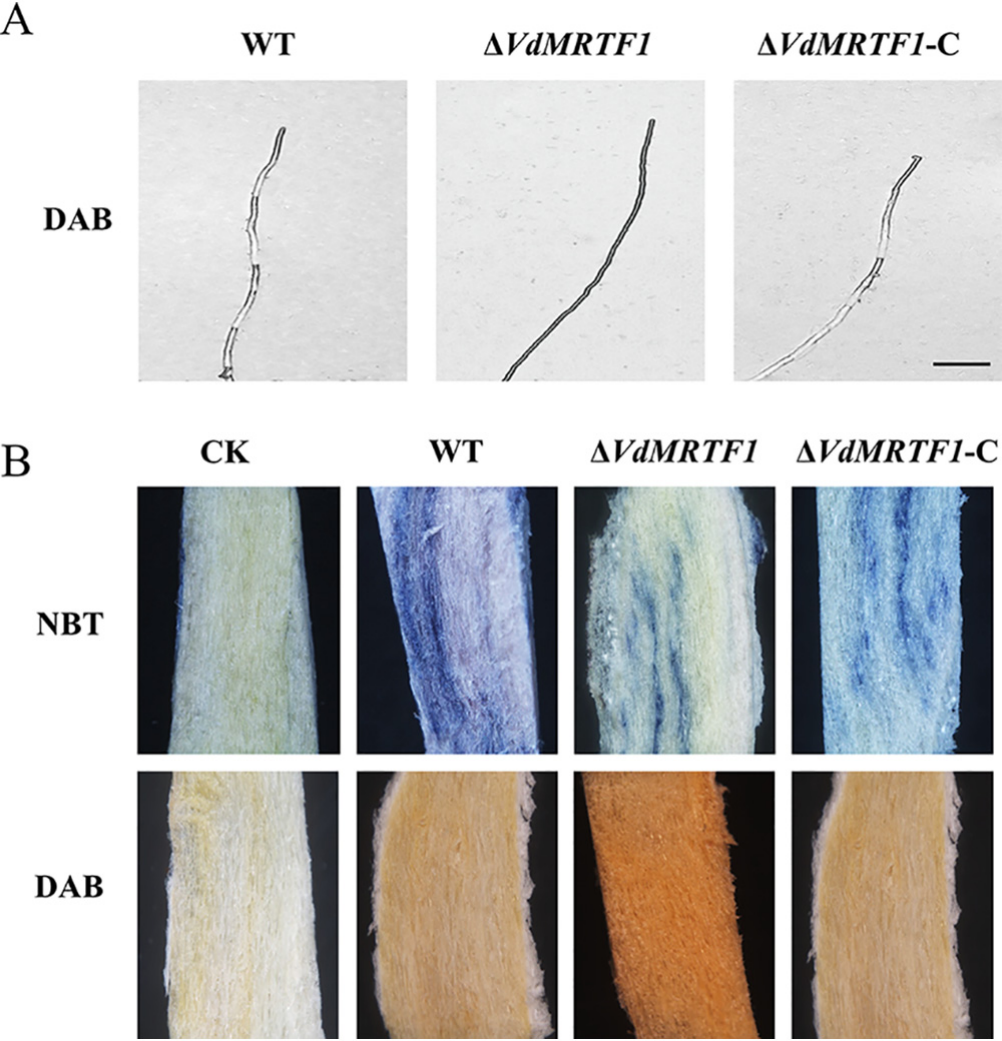
VdMRTF1 is involved in eliminating endogenous and exogenous reactive oxygen species *in* Verticillium dahliae. (A) The hyphae of all stains were stained with diaminobenzidine 3,3′-diaminobenzidine (DAB) overnight and observed by light microscopy (DM2500, Leica). Bar = 20 μm. (B) Tobacco seedling roots infected with the wild-type, Δ*VdMRTF1*, and Δ*VdMRTF1*-C strains at 7 dpi. Controls (CK) were mock-inoculated with distilled water. The nitroblue tetrazolium (NBT) and DAB stains were used to visualized O_2_.- and H_2_O_2_, respectively.

Due to a fundamental role of reactive oxygen species (ROS) in plant-fungal interactions (18), we assessed whether deletion of *VdMRTF1* influenced the ability of V. dahliae to scavenge hydrogen peroxide (H_2_O_2_) and O_2_- produced by plants. Both of nitroblue tetrazolium (NBT) and 3,3′-diaminobenzidine (DAB) staining were used to analyze the accumulation of O_2_- and H_2_O_2_, respectively. To investigate whether VdMRTF1 has the ability to eliminate endogenous ROS, the hyphae of all strains were immersed in DAB staining to analyze the accumulation of peroxidase. The hyphae of the Δ*VdMRTF1* strain showed deeper reddish-brown spots than that observed for the wild-type strain and the Δ*VdMRTF1*-C strain showed a similar level of staining as the wild type (Fig. 5A). Additionally, roots of tobacco seedlings challenged with V. dahliae strains were collected at 7 dpi, and tobacco seedlings roots inoculated with the Δ*VdMRTF1* stain showed light blue spots following NBT staining and deep reddish-brown spots following DAB staining. In contrast, roots of tobacco seedlings inoculated with the wild-type strain showed deep blue spots following NBT staining and light reddish-brown spots by DAB staining. Roots of tobacco seedlings inoculated with the complementation strain showed NBT and DAB staining patterns similar to the wild type (Fig. 5B). These results suggested that VdMRTF1 is involved in eliminating exogenous ROS produced by host plants and endogenous ROS produced by V. dahliae.

### RNA-seq analyses of the Δ*VdMRTF1* strain of V. dahliae.

To investigate how VdMRTF1 regulates melanin biosynthesis, we performed comparative RNA-seq with the Δ*VdMRTF1* and wild-type strains and examined the differentially expressed genes (DEGs) in each. Because previous results showed that the Δ*VdMRTF1* strain formed melanized microsclerotia while the wild-type and the *VdMRTF1* complemented strain did not form melanized microsclerotia in liquid BM medium, we gathered microsclerotia cultured in liquid BM mediums after 10 days for the RNA-seq experiments. RNA-seq analysis revealed 317 DEGs between the wild-type and the Δ*VdMRTF1* strains in total, including 178 upregulated and 139 downregulated genes with fold change ≥1.5 (Fig. S3A). Among the top 10 upregulated DEGs, six DEGs encoded products with putative enzyme activity, one DEG was predicted to have manganese ion transmembrane transporter activity, and the rest of DEGs were uncharacterized proteins. Among the top 10 downregulated DEGs, only one DEG encoded a product with predicted enzyme activity, one DEG in chitin binding, one DEG with predicted catalytic activity, and the rest of DEGs were uncharacterized proteins (Table S2).

**FIG 6:**
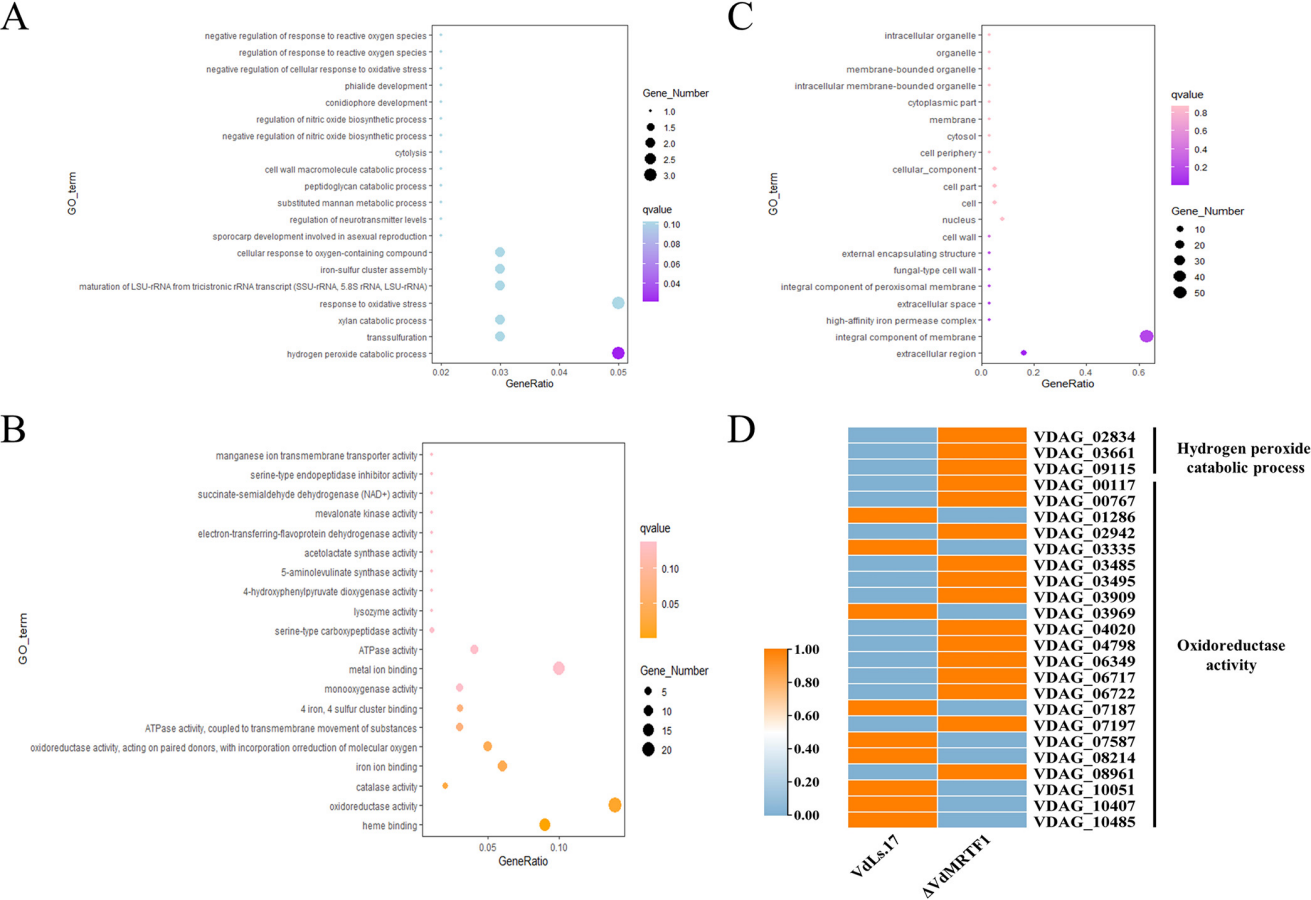
The gene ontology (GO) enrichment analysis of DEGs in wild-type and *ΔVdMRTF1* strains of Verticillium dahliae. (A) Bubble chart showing DEGs enriched in biological process of GO terms. The size of the bubbles represents the gene number of DEGs of each GO term. The *x* axis indicates the gene ratio, and the *y* axis indicates the GO terms. (B) Bubble chart showing DEGs enriched in molecular function of GO terms. The size of the bubbles represents the gene number of DEGs of each GO term. The *x* axis indicates the gene ratio, and the *y* axis indicates the GO terms. (C) Bubble chart showing DEGs enriched in cellular component of GO terms. The size of the bubbles represents the gene number of DEGs of each GO term. The *x* axis indicates the gene ratio, and the *y* axis indicates the GO terms. (D) Heatmap of DEGs in hydrogen peroxide catabolic process and oxidoreductase activity. The date was based on the average FPKM value of three biological repetitions, and TBtools was used to normalize data and generate heat maps. The orange color indicates relatively higher FPKM value, while the light blue color indicates relatively lower FPKM value.

To further probe the function of VdMRTF1 in V. dahliae, we examined the gene ontology (GO) results in more detail in relation to the RNA-seq data. The GO analyses of the DEGs revealed that the top three GO terms of upregulated and downregulated DEGs are the same, including catalytic activity, metabolic process and binding (Fig. S4A, B). The top 20 GO enriched terms under biological process, molecular function, and cellular component are shown by bubble charts. The bubble chart of biological process illustrated that the DEGs were enriched in hydrogen peroxide catabolic process (GO:0042744), transsulfuration (GO:0019346), xylan catabolic process (GO:0045493), response to oxidative stress (GO:0006979), maturation of LSU-rRNA from tricistronic rRNA transcript (SSU-rRNA, 5.8S rRNA, LSU-rRNA) (GO:0000463), iron-sulfur cluster assembly (GO:0016226), and cellular response to oxygen-containing compound (GO:1901701) (Fig. 6A). The bubble chart for molecular function showed that the DEGs were enriched in heme binding (GO:0020037), oxidoreductase activity (GO:0016491), catalase activity (GO:0004096), iron ion binding (GO:0005506), and oxidoreductase activity, acting on paired donors, with incorporation or reduction of molecular oxygen (GO:0016705) (Fig. 6B). The bubble chart of cellular component revealed that the DEGs were enriched in extracellular region (GO:0005576) and integral component of membrane (GO:0016021) (Fig. 6C). The results above suggested that VdMRTF1 plays a role in the regulation of some of the genes involved in eliminating ROS, consistent with findings above. Therefore, we specifically examined the expression of genes involved in hydrogen peroxide and oxidoreductase activity. A heatmap of DEGs involved in hydrogen peroxide catabolic process (GO:0042744) and oxidoreductase activity (GO:0016491) was prepared based on fragments per kilobase of exon model per million mapped fragments (FPKM). RNA-seq data revealed that the expression of all DEGs enriched in hydrogen peroxide catabolic process (VDAG_02834, VDAG_03661, and VDAG_09115) were upregulated in the Δ*VdMRTF1* strain. The expression of most of DEGs (VDAG_00117, VDAG_00767, VDAG_02942, VDAG_03485, VDAG_03495, VDAG_03909, VDAG_04020, VDAG_04798, VDAG_06349, VDAG_06717, VDAG_06722, VDAG_07197, and VDAG_08961) enriched in oxidoreductase activity were upregulated, the expression of the rest of DEGs (VDAG_01286, VDAG_03335, VDAG_03969, VDAG_07187, VDAG_07587, VDAG_08214, VDAG_10051, VDAG_10407, and VDAG_10485) were downregulated (Fig. 6D). These results indicated that *VdMRTF1* disrupted the expression of genes involved in oxidoreductase activity and hydrogen peroxide catabolic process to eliminate ROS.

### VdMRTF1 regulates expression of genes involved in melanin biosynthesis.

**FIG 7:**
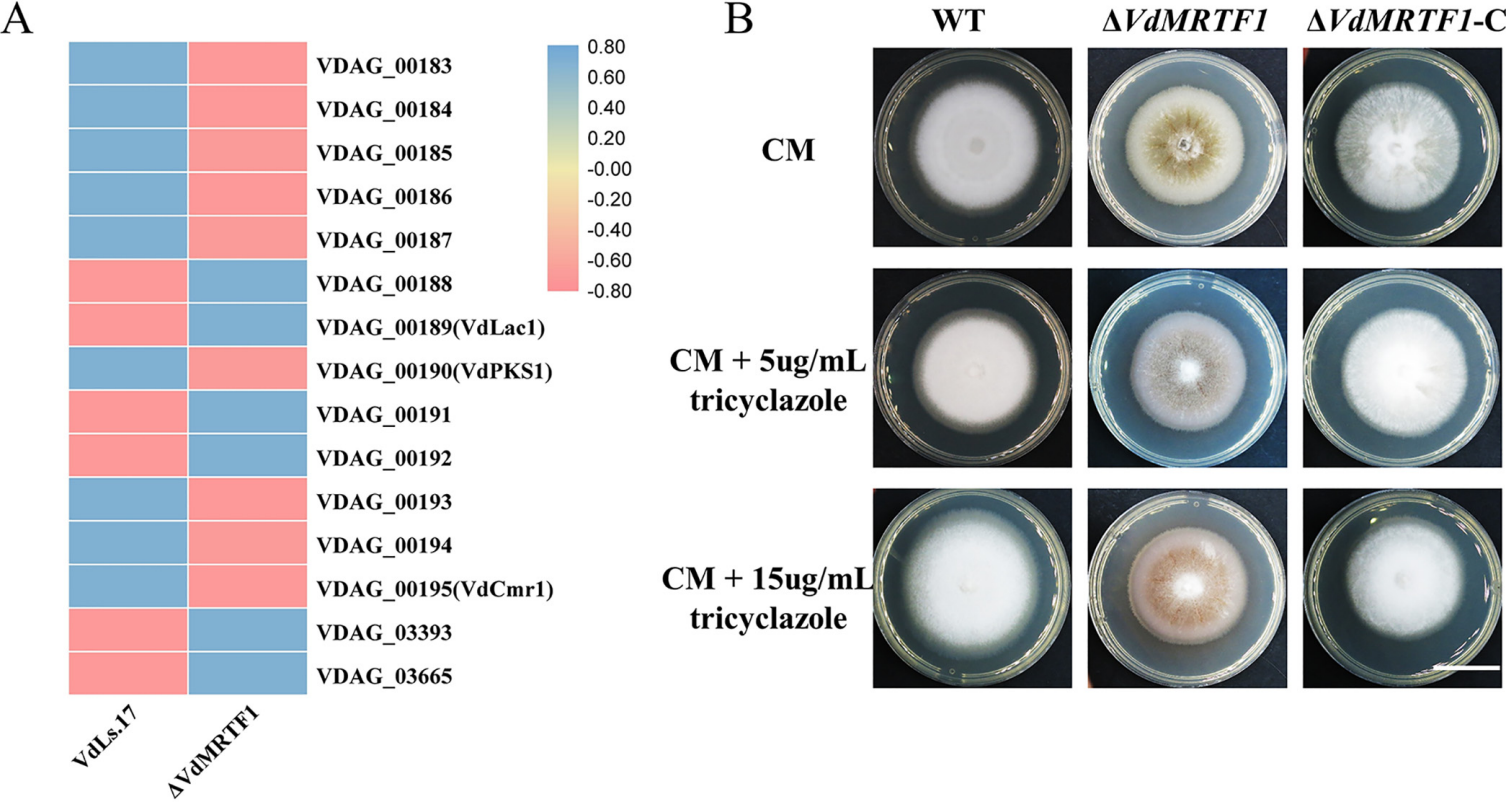
*VdMRTF1* regulates expression of genes involved in melanin biosynthesis in Verticillium dahliae. (A) Heatmap indicating the FPKM value of genes in a secondary metabolism gene cluster, which are involved in melanin biosynthesis in V. dahliae. The average FPKM value was from three biological repetitions. TBtools was used to normalize data and generate a heat map. The pink color indicates expression was relatively higher in the Δ*VdMRTF1* compared with the wild-type VdLs.17 strain, while the blue color indicates expression was relatively lower. (B) The wild-type, *VdMRTF1* mutant, and complemented strains were cultured in complete medium (CM), CM with 5 μg/mL, and CM with 15 μg/mL tricyclazole at 25°C for 10 days. Photographs were taken at 10 dpi. Bar, 2 cm.

Previous research has revealed involvement of a number of genes in DHN-melanin synthesis in V. dahliae (6, 34). Because deletion of *VdMRTF1* in V. dahliae results in increased melanin biosynthesis, we generated a heatmap of genes involved in melanin biosynthesis by FPKM. Transcripts of VDAG_00188 (hypothetical protein), VDAG_00189 (laccase, *VdLac1*), VDAG_00191 (hypothetical protein), VDAG_00192 (putative transcription factor), VDAG_03393 (scytalone dehydratase), and VDAG_00365 (THN reductases) were upregulated while the expression of VDAG_00183 (THN-reductase), VDAG_00184 (polyketide synthase, *PKS*), VDAG_00185 (hypothetical protein), VDAG_00186 (pyridoxal-dependent decarboxylase), VDAG_00187 (DUF92 domain containing protein), VDAG_00190 (polyketide synthase, *PKS*), VDAG_00193 (hypothetical protein), VDAG_00194 (Pig1 transcription factor), and VDAG_00195 (*Cmr1* transcription factor) were downregulated (Fig. 7A). To examine further whether VdMRTF1 has the ability to influence DHN-melanin production, tricyclazole (an inhibitor of DHN-melanin biosynthesis) was applied. The results showed that the Δ*VdMRTF1* strain formed less melanin when 5 μg/mL tricyclazole or 15 μg/mL tricyclazole was blended with complete medium (CM) while there was no difference of that in the wild-type and the complemented strains (Fig. 7B). Taken together, these results indicated that VdMRTF1 regulated expression of genes involved in melanin biosynthesis.

### VdMRTF1 regulates tyrosine metabolism.

**FIG 8:**
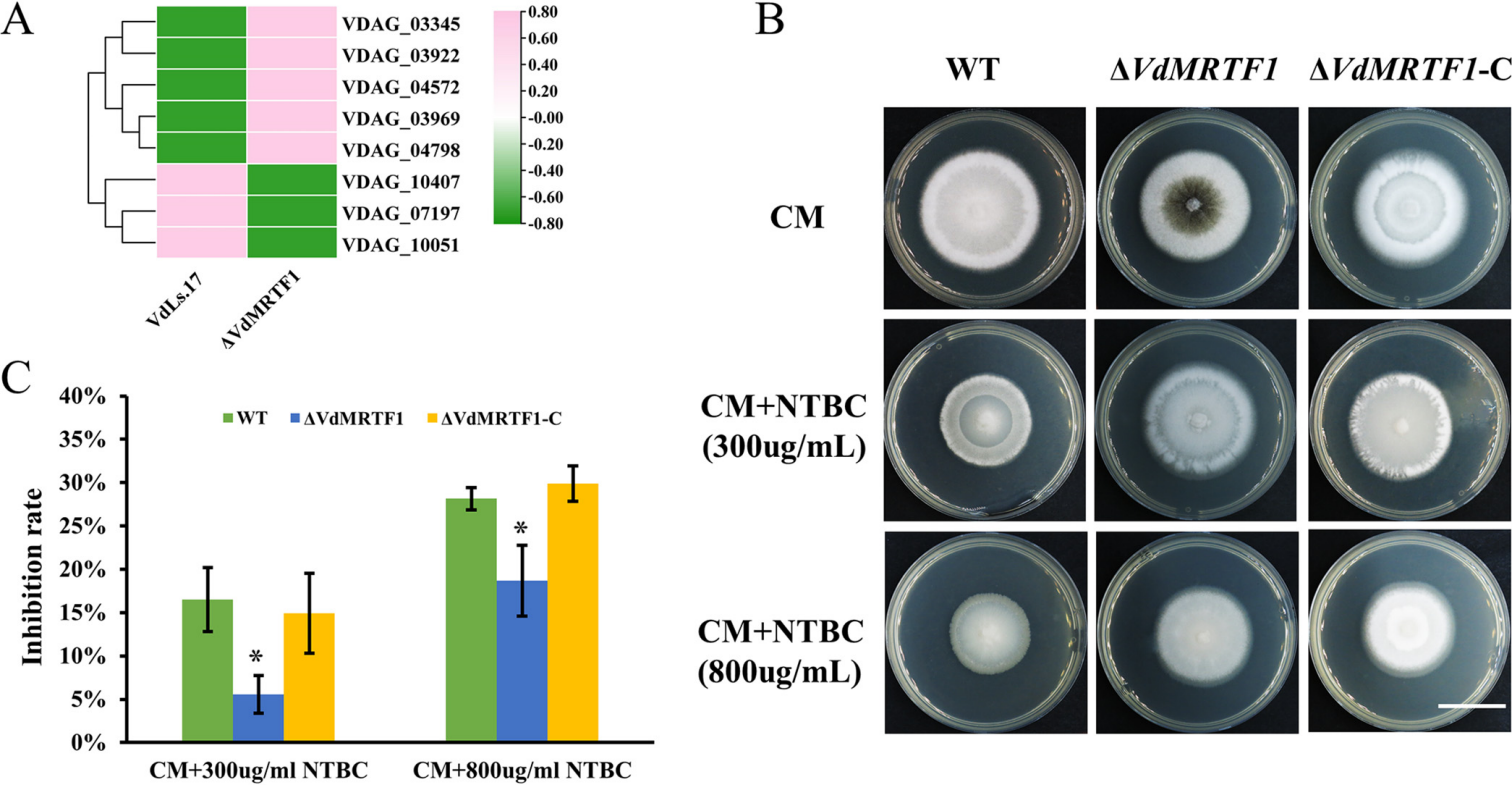
VdMRTF1 regulates tyrosine metabolism *in* Verticillium dahliae. (A) Heatmap of DEGs in tyrosine metabolism enriched in the KEGG pathway analysis. TBtools was used to normalize data and generate a heat map based on the average FPKM value of three biological repetitions. The pink color indicates relatively higher FPMK, while the green color indicates relatively lower FPMK. (B) The wild-type, Δ*VdMRTF1*, and Δ*VdMRTF1*-C strains were cultured in CM, CM with 300 μg/mL, and CM with 800 μg/mL NTBC 2-(2-nitro4-trifluoromethylbenzoyl)-cyclohexane-1, 3-dione) at 25°C for 10 days. Photographs were taken at 10 dpi. Bar, 2 cm. (C) Bar chart of the inhibition rate of the above-described plates. Error bars represent the standard deviations based on three independent replicates. Asterisks indicate significant differences (*P < 0.5).

To further investigate the function of VdMRTF1 in V. dahliae, we performed Kyoto Encyclopedia of Genes and Genomes (KEGG) enrichment of all DEGs and classified the top 20 KEGG enrichment terms in a histogram. The DEGs were mostly distributed in the following KEGG pathways: tyrosine metabolism (KO00350), biosynthesis of amino acids (KO01230), butanoate metabolism (KO00650), ATP-binding cassette (ABC) transporters (KO02010), tryptophan metabolism (KO00380), peroxisome (KO04146) and glycine, serine, and threonine (KO00260) (Fig. S5A). As show in the bubble chart based on enrichment factor, q-value, and gene number of each KEGG pathway, the most reliable KEGG enrichment pathway is tyrosine metabolism (KO00350), for which the q-value of tyrosine metabolism is the lowest (Fig. S5B). Among eight DEGs involved in this KEGG pathway, five DEGs (VDAG_03345, VDAG_03922, VDAG_04572, VDAG_03969, and VDAG_04798) were significantly upregulated, while the remaining DEGs (VDAG_10407, VDAG_07197, and VDAG_10051) were significantly downregulated (Fig. 8A). To verify whether VdMRTF1 is involved in tyrosine metabolism, NTBC (an inhibitor of toxic 4-hydroxyphenylpyruvate, which is on the second step of tyrosine metabolism) was used to inhibit tyrosine metabolism of V. dahliae growing on CM. Colonies of the wild-type strain were notably reduced in size compared with the Δ*VdMRTF1* strain on CM containing 300 μg/mL and 800 μg/mL NTBC. The inhibition rate of the Δ*VdMRTF1* strain cultured in CM containing 300 μg/mL NTBC (5.6% for the Δ*VdMRTF1* strain; 16.5% for the wild-type strain) or 800 μg/mL NTBC (18.7% for the Δ*VdMRTF1* strain; 28.2% for the wild-type strain) was significantly lower than that of the wild-type strain. The Δ*VdMRTF1*-C strain showed an inhibition rate comparable to that of the wild-type strain (Fig. 8B and C). These results suggested that VdMRTF1 plays a role in tyrosine metabolism and functions in detoxification.

## DISCUSSION

One of our previous works had indicated that the transcription factor VdMRTF1 may participate in microsclerotia formation (35). In this study, we generated a *VdMRTF1* mutant strain and the corresponding complemented mutant strain to test the hypothesis that VdMRTF1 regulates microsclerotia production. In the conduction of these experiments, we found that VdMRTF1 negatively regulates microsclerotia formation and melanin biosynthesis, and deletion of *VdMRTF1* reduces microsclerotia germination in V. dahliae. The RNA-seq data indicated that VdMRTF1 disrupts the expression of genes involved in DHN-melanin biosynthesis, and VdMRTF1 modulated sensitivity to tricyclazole. Additionally, VdMRTF1 is involved in resistance to stress conditions such as elevated Ca^2+^. We found that the Δ*VdMRTF1* strain showed increased virulence in host plants relative to the wild-type strain. Given that ROS plays an important role in plant-fungus interactions (36) and VdMRTF1 has the ability to eliminate endogenous and exogenous ROS, the observed increased virulence this may be mediated by indirect action of VdMRTF1 in eliminating ROS. Indeed, the results from the RNA-seq experiments showed that *VdMRTF1* regulates the expression of genes involved in oxidoreductase activity and the hydrogen peroxide catabolic process. Finally, RNA-seq analyses suggested that VdMRTF1 may have a role in tyrosine metabolism; the expression of genes involved in tyrosine metabolism were disrupted by VdMRTF1, and VdMRTF1 had the ability to detoxicate of 4-hydroxyphenylpyruvate metabolized by tyrosine.

Melanized microsclerotia play a critical role in the disease cycle of V. dahliae, allowing this fungus to survive more than 10 years in the soil, even without a host (2). Once environmental conditions are suitable, microsclerotia may geminate and infect host plants (4, 35). Due to the importance in the disease cycle of V. dahliae, there has been research directed at determining the genetic and biochemical bases regulating microsclerotia formation in this fungus (12). For instance, deletion of transcription factors VdSkn7, VdHapX, or VdYap1 reduce microsclerotia formation, while deletion of VdMsn2 enhanced microsclerotia formation in V. dahliae (17, 18, 34, 37). Here, we have shown that the transcription factor VdMRTF1 negatively regulates the formation of melanized microsclerotia.

Mature microsclerotia of wild-type V. dahliae consist of many melanized cells (38), though melanization can be uncoupled from microsclerotia formation since genetic mutants of melanin biosynthetic genes can form microsclerotia devoid of pigment (6). Considering that the Δ*VdMRTF1* strain exhibited increased melanin biosynthesis, we observed the formation of microsclerotia in both the wild-type and *VdMRTF1* mutant strains (21). Intriguingly, in this study, the Δ*VdMRTF1* strain formed more melanin than the wild-type VdLs.17 strain, both on PDA and on BM plates. We also cultured the Δ*VdMRTF1*, the wild-type VdLs.17, and the Δ*VdMRTF1*-C strains in liquid BM medium, and the result was the same as on BM plates. The micrograph showed that in comparison with the wild strain, the Δ*VdMRTF1* strain formed much higher numbers of microsclerotia, though deletion of VdMRTF1 did not affect the morphology of the microsclerotia.

Many studies have revealed a linkage between melanin and virulence in fungi (11, 12, 14, 39, 40). DHN-melanin is essential for host penetration in *M. grisea*, as it provides the rigidity of appressoria to penetrate plant leaves (30). Some species of the *Sporothrix* complex were able to produce pyomelanin which may act as a pathogenicity factor (19). Aspergillus fumigatus can produce two types of melanin: DHN-melanin and pyomelanin, and the DHN-melanin was confirmed to eliminate ROS produced by host plants thereby providing protection against ROS (28). Besides protecting A. fumigatus against the host’s immune defenses, defects in DHN-melanin production may disrupt intracellular signaling and metabolic pathways and influences the proper function of adhesins and hydrophobins (28, 41). In our study, the results of NBT and DAB staining illustrated that deletion of *VdMRTF1* led to increased elimination of ROS produced by host plants. Both RNA-seq and the results of sensitivity tests to tricyclazole revealed that gene expression and biological processes involved in DHN-melanin biosynthesis were disrupted in the Δ*VdMRTF1* mutant. Given these results, we speculate that deletion of *VdMRTF1* increases virulence through eliminating ROS produced by host plants, and regulates genes involved in melanin biosynthesis to accelerate DHN-melanin biosynthesis.

In this study, RNA-seq was used to further investigate the function of VdMRTF1 of regulating gene expression in V. dahliae. RNA-seq analyses indicated that 178 genes were upregulated and 139 genes were downregulated in the wild-type VdLs.17 compared with the Δ*VdMRTF1* strain after culturing in BM medium for 10 days. Based on GO enrichments, we found that most of DEGs were enriched in catalytic activity, metabolic process, or binding. Moreover, DEGs involved in hydrogen peroxide catabolic process were all upregulated. ROS play a foundational role in plant-fungal interactions. As an integral part of the defense of plants, ROS were consistently observed to accumulate in the plant during plant-V. dahliae interactions and hence detoxification of host-derived ROS may be required for increased fungal virulence. The forms of ROS include superoxide anion (O_2_-), hydrogen peroxide (H_2_O_2_), hydroxyl radical (OH), or hydroperoxyl radical (HO_2_) (42). The RNA-seq data suggested that some genes participate in hydrogen peroxide catabolic process, such as VDAG_02834, VDAG_03661, and VDAG_09115 which were upregulated. The RNA-seq data also supported results obtained by NBT and DAB staining of infected tobacco seedlings and hyphae. Genes encoding products with oxidoreductase activity were also differentially expressed in the Δ*VdMRTF1* strain. Among 22 DEGs involved in oxidoreductase activity, 13 of DEGs were upregulated and the remaining were downregulated, providing additional evidence for the function of VdMRTF1 in eliminating ROS during plant-fungal interactions. Taken together, we concluded that observed increased virulence of the *VdMRTF1* mutant might be explained by altered expression of genes whose products participate in hydrogen peroxide catabolic processes or oxidoreductase activity.

Based on KEGG enrichment, we found that many DEGs observed in the Δ*VdMRTF1* strain were enriched in tyrosine metabolism, butanoate metabolism or transport (ABC transporters specifically). Tyrosine metabolism is related to the biosynthesis of melanin and can be utilized as a nutrient source. Some species of *Sporothrix* complex were able to produce pyomelanin or DHN-melanin when provided tyrosine (19). In addition to DHN-melanin, Alternaria alternata produces another type of melanin-pyomelanin, which is derived from l-tyrosine (43). In A. fumigatus, the biosynthesis of pyomelanin is related to the activation of the l-tyrosine or l-phenylalanine degradation pathway (28). Tyrosine is a pivotal nutrient source during infectious growth in fungal pathogen *Penicillium marneffei* (44). Similarly, our RNA-seq data analyses suggested that the genes involved in tyrosine metabolism were differently expressed in the Δ*VdMRTF1* strain of V. dahliae. The intermediate products 4- hydroxyphenylpyruvate are produced in the tyrosine catabolism pathway, which is toxic (44–46). Among DEGs enriched in tyrosine metabolism, VDAG_03922 was upregulated and has the predicted function of regulating 4-hydroxyphenylpyruvate dioxygenase activity. We speculated that upregulation of VDAG_03933 may help to catabolize toxic 4-hydroxyphenlpyruvate and thereby protect the Δ*VdMRTF1* mutant strain.

To determine whether VdMRTF1 is involved in tyrosine metabolism, an inhibitor of tyrosine metabolism NTBC was added in CM medium. The wild-type strain showed obvious reduction in growth relative to the Δ*VdMRTF1* strain in CM medium supplemented with different concentrations of NTBC. From this result, we drew the conclusion that VdMRTF1 is involved in tyrosine metabolism and participated in detoxication. Additionally, DEGs in the Δ*VdMRTF1* mutant strain relative to the wild-type VdLs.17 were also enriched in ABC transporters according to KEGG enrichment, and these play important roles in transporting a wide variety of compounds across biological membranes through ATP hydrolysis (47). They are also involved in drug detoxification in yeasts, and some of the ABC transporters participate in fungal virulence (48, 49). Taken together, analyses of the RNA-seq data revealed that VdMRTF1 regulates the expression of genes involved in tyrosine metabolism and those encoding ABC transporters.

In summary, the results of this study indicate that the transcription factor VdMRTF1 plays important roles in melanin biosynthesis, microsclerotia formation, and virulence in plant pathogenetic fungus V. dahliae. The RNA-seq data revealed that VdMRTF1 contributes to melanin biosynthesis and virulence possibly by regulating the expression of genes involved in DHN-melanin biosynthesis, tyrosine metabolism, hydrogen peroxide catabolic process, and oxidoreductase activity. The results of NBT and DAB staining revealed that VdMRTF1 has the ability to eliminate ROS produced by host plants. The results of sensitivity tests to NTBC demonstrated VdMRTF1 participates in tyrosine metabolism. The data presented in this study provide new insight on how a bZip transcription factor functions in the regulation of melanin biosynthesis, microsclerotia formation, and virulence. VdMRTF1 is a potential target to control microsclerotia formation and virulence in V. dahliae.

## MATERIALS AND METHODS

### Fungal strains and culture conditions.

The V. dahliae strain VdLs.17 isolated from lettuce (50) was used as parental strain to generate gene deletion mutants in this study. Unless specified otherwise, all strains in this study were initially incubated on PDA medium (200 g potato, 20 g glucose, 15 g agar per L) at 25°C. Conidia used for all experiments were acquired from a colony that was incubated for 7 days on PDA medium.

To observe melanin biosynthesis and microsclerotia formation, 1 mL of a conidial suspension of V. dahliae (10^5^/mL) was coated on a cellulose membrane (Ø = 80 mm; pore size = 0.22 μm), which was overlaid on solid basal medium (10g glucose, 0.2g NaNO_3_, 0.52g KCl, 1.52g KH_2_PO_4_, 0.52g MgSO_4_·7H_2_O, 3 μmol thiamine HCl, 0.1umol biotin,15 g agar per L) and then cultured at 25°C. The observation of melanin formation was documented by photography after 4, 7, and 14 days; and the melanized area fraction was measured by ImageJ (15). The microsclerotia formation was performed by light microscopy (DM2500, Leica) and field emission scanning electron microscope (SU8010) after 4, 7, and 14 days. The number of microsclerotia on cellulose membrane (per 8.8 square millimeter) was calculated by ImageJ (15). To test the germination rate of microsclerotia, 14-day-old microsclerotia of all strains were collected from a cellulose membrane. Sifters with 125 μm pores and 38 μm pores were used to isolate microsclerotia. Microsclerotia were collected from the 38 μm sifter and incubated at 47°C for 5 min to kill hypha and conidia (51). One-hundred single microsclerotia of each strain were inoculated on PDA at 25°C and photographs were taken after 7 days, then the photographs were removed to ImageJ to calculate the number of germinated microsclerotia of each strain. To test resistance to temperature extremes, microsclerotia of all strains were stored at −40°C for 48 h, and then 100 single microsclerotia of each strain were inoculated on PDA at 25°C and photographs and quantification were taken after 7 days as before. To observe melanin formation in liquid BM, a 1 mL conidial suspension (10^5^/mL) of each strain was added into liquid BM and shaken (150 rpm, 25°C) for 10 days, and then documented by photography. All experiments were repeated at least 3 times.

For resistance to Ca^2+^ assays, all strains were cultured in PDA containing different concentration of CaCl_2_, including 0.4 M CaCl_2_ and 0.6 M CaCl_2_ at 25°C for 10 days, and photographs were taken. All experiments were repeated 3 times.

To test the response to tricyclazole, all strains were cultured for 10 days on CM (50 mL of 20 nitrate salts, 1 mL 1,000× trace elements, 10 g glucose, 2 g peptone, 1 g yeast extract, 1 g Casamino Acids, 1 mL vitamin solution per L) amended with 5 μg/mL, and 15 μg/mL tricyclazole. All plates were incubated at 25°C and photographs were taken after 10 days. All experiments were repeated at least 3 times.

To investigate tyrosine metabolism, the chemical inhibitor 2-[2-nitro-4-(trifluoromethyl) benzoyl]-1, 3-cyclohexanedione (NTBC, Shanghai yuanye Bio-Technology, Shanghai, China) of tyrosine metabolism was used in this study. The wild-type, Δ*VdMRTF1*, and Δ*VdMRTF1*-C strains were cultured in CM medium, CM with 300 μg/mL NTBC, and CM with 800 μg/mL NTBC for 10 days at 25°C and photograph were taken. The experiments were repeated 3 times.

### Deletion and complementation of *VdMRTF1* in V. dahliae.

To delete *VdMRTF1* in the wild-type strain VdLs.17, the split-marker method (52) was used for generating the two deletion constructs, the 5F and the 3F deletion cassettes. Firstly, the primer pairs bZIP255Fs/bZIP255Fx and bZIP253Fs/bZIP253Fx were used to get 5F and 3F flanking sequences by PCR respectively. Then, the hygromycin resistance cassettes was amplified with primer pair HYF/HYR so that it contained approximately 20 bp overlaps with the 5F and the 3F flanking sequences. Subsequently, both flanking sequences were fused to the hygromycin resistance by fusion PCR. Finally, the overlapping DNA fragments verified by sequencing were used directly to protoplast transformation (11). The primer pairs bZIP255ns/bZIP255nx and bZIP25zhs/bZIP25zhx were used to screen the *VdMRTF1* mutants through PCR amplification. Southern blotting was performed with the DIG High Prime DNA Labeling and Detection Starter Kit I in accordance with the manufacturers’ protocol (Roche, Germany). The primer pairs P123/P124 were used to amplify the probe fragment labeled with DIG primer. The restriction enzyme KpnI was used to digest genomic DNA extracted from the wild-type, Δ*VdMRTF1* and Δ*VdMRTF1*-C strains.

To complement the Δ*VdMRTF1* strain, the primer pairs bZIP25hbs/bZIP25hbx were used to amplify the fragment containing the coding sequence of wild-type *VdMRTF1* without the termination codon. All primers used in this study are listed in Table S1.

### Phylogenetic analysis.

Protein sequences of VdMRTF1 and its homologs was downloaded from NCBI (https://www.ncbi.nlm.nih.gov/). The amino acid sequence alignments were performed with ClustalX (53). The phylogenetic tree was constructed with MEGAX using the neighbor joining algorithm and the bootstrap test was replicated 1,000 times (54).

### Plant infection assays.

The wild-type, Δ*VdMRTF1*, and Δ*VdMRTF1*-C strains were cultured in liquid CM medium for 5 days, the conidia were harvested by filtration through two layers of Miracloth (Millipore), and adjusted to 10^6^ conidia/mL in sterile water. Thirty 1-month-old tobacco seedlings (Nicotiana benthamiana) were used for virulence assays for each strain. The roots of tobacco seedlings were dipped in conidial suspensions for 10 min and all plants were replanted into autoclaved sterile soil(11). Control plants were mock inoculated with distilled water. All tobacco seedlings were placed in a greenhouse at 25°C and observed over a period of 35 days. The experiments were repeated 3 times.

### Histochemical detection of superoxide.

The infected roots of tobacco seedings were collected for analyses of ROS detection. Both of the nitroblue tetrazolium (NBT, Solarbio, Beijing, China) and 3,3′-diaminobenzidine (DAB, Coolaber, Beijing, China) staining were used to detect ROS (42, 55). The roots of tobacco seedings infected with the wild-type, the *ΔVdMRTF1* strain, and the complemented strains were collected at 7 dpi, and roots were immersed in either 0.05% NBT or 0.1% DAB staining solution. All roots were saved in the tubes and kept overnight in dark. Roots were immersed in absolute ethanol and heated in boiling water bath for 10 min to completely eliminate the chlorophyll. Superoxide production was visualized as a purple formazan deposit within roots tissues by stereo microscope (M205FA, Leica).

The 3-day-old hyphae of the wild-type, Δ*VdMRTF1*, and Δ*VdMRTF1*-C strains were immersed in 0.1% DAB staining solution overnight in dark. Then, the chromatic hyphae were observed by light microscopy (DM2500, Leica).

### RNA extraction and RNA-sequencing.

The 1-mL conidial suspension (10^7^ spores/mL) of the wild-type, Δ*VdMRTF1*, and complemented strains was added into 150 mL liquid BM in shake culture (150 rpm, 25°C) for 10 days. The microsclerotia of each strain were collected and send to Biomarker Technologies (Beijing, China) for RNA extraction and RNA-seq. There were three biological replicates using each strain.

### DEGs analysis, GO analysis, and KEGG analysis.

The edgeR (56) was used to determine DEGs between the wild-type and the *VdMRTF1* mutant strain. Those genes with fold change ≥1.5 and *P*-value < 0.01were considered significant DEGs. Differentially expressed genes were functionally classified according to the GO annotations results (http://geneontology.org/), and the software package “ggplot” in R was used for enrichment analysis. Based on KEGG annotation results (https://www.genome.jp/kegg/), the DEGs were classified into biological pathways using the same methods as GO analysis. Heat maps of expression values were normalized and made by TBtools (57).

### Statistical analysis.

Data were expressed as mean value ± standard error of the mean and analyzed with a one-way ANOVA independent-samples Tukey’s range test using SPSS for Windows version 16.0 (Chicago, IL, USA). The *P*-value < 0.5 was considered statistically significant, and asterisks are used to indicate *P* < 0.5 (*) and *P* < 0.001 (***).

### Data availability.

The raw sequence and other related data reported in this paper have been deposited in the BIG Data Center, Chinese Academy of Sciences (https://ngdc.cncb.ac.cn/). The accession number of the transcriptomes is CRA005983 (https://ngdc.cncb.ac.cn/gsa/browse/CRA005983).

## References

[1] Klosterman SJ, Atallah ZK, Vallad GE, Subbarao KV. 2009. Diversity, pathogenicity, and management of *Verticillium* species. Annu Rev Phytopathol 47:39–62. doi:10.1146/annurev-phyto-080508-081748. 19385730

[2] Bhat RG, Subbarao KV. 1999. Host range specificity in *Verticillium dahliae*. Phytopathology 89:1218–1225. doi:10.1094/PHYTO.1999.89.12.1218. 18944648

[3] Wang Y, Wang Y, Tian C. 2013. Quantitative detection of pathogen DNA of Verticillium wilt on smoke tree *Cotinus coggygria*. Plant Dis 97:1645–1651. doi:10.1094/PDIS-04-13-0406-RE. 30716826

[4] Schnathorst WC. 1981. Life cycle and epidemiology of *Verticillium*. Fungal Wilt Diseases of Plants 82:81–111.

[5] Garas NA, Wilhem S, Sagen JE. 1986. Relationship of cultivar resistance to distribution of *Verticillium dahliae* in inoculated cotton plants and to growth of single conidia on excised stem segments. Phytopathology 76:1005–1010. doi:10.1094/Phyto-76-1005.

[6] Wang Y, Hu X, Fang Y, Anchieta A, Goldman PH, Hernandez G, Klosterman SJ. 2018. Transcription factor VdCmr1 is required for pigment production, protection from UV irradiation, and regulates expression of melanin biosynthetic genes in *Verticillium dahliae*. Microbiology (Reading) 164:685–696. doi:10.1099/mic.0.000633. 29485393 PMC5982140

[7] Klimes A, Dobinson KF, Thomma BP, Klosterman SJ. 2015. Genomics spurs rapid advances in our understanding of the biology of vascular wilt pathogens in the genus *Verticillium*. Annu Rev Phytopathol 53:181–198. doi:10.1146/annurev-phyto-080614-120224. 26047557

[8] Chen JY, Klosterman SJ, Hu XP, Dai XF, Subbarao KV. 2021. Key insights and research prospects at the dawn of the population genomics era for *Verticillium dahliae*. Annu Rev Phytopathol 59:31–51. doi:10.1146/annurev-phyto-020620-121925. 33891830

[9] Xiong D, Wang Y, Ma J, Klosterman SJ, Xiao S, Tian C. 2014. Deep mRNA sequencing reveals stage-specific transcriptome alterations during microsclerotia development in the smoke tree vascular wilt pathogen, *Verticillium dahliae*. BMC Genomics 15:324. doi:10.1186/1471-2164-15-324. 24884698 PMC4035056

[10] Duressa D, Anchieta A, Chen D, Klimes A, Garcia-Pedrajas MD, Dobinson KF, Klosterman SJ. 2013. RNA-seq analyses of gene expression in the microsclerotia of *Verticillium dahliae*. BMC Genomics 14:607. doi:10.1186/1471-2164-14-607. 24015849 PMC3852263

[11] Xiong D, Wang Y, Tang C, Fang Y, Zou J, Tian C. 2015. *VdCrz1* is involved in microsclerotia formation and required for full virulence in *Verticillium dahliae*. Fungal Genet Biol 82:201–212. doi:10.1016/j.fgb.2015.07.011. 26235044

[12] Li JJ, Zhou L, Yin CM, Zhang DD, Klosterman SJ, Wang BL, Song J, Wang D, Hu XP, Subbarao KV, Chen JY, Dai XF. 2019. The *Verticillium dahliae* Sho1-MAPK pathway regulates melanin biosynthesis and is required for cotton infection. Environ Microbiol 21:4852–4874. doi:10.1111/1462-2920.14846. 31667948 PMC6916341

[13] Wang Y, Tian L, Xiong D, Klosterman SJ, Xiao S, Tian C. 2016. The mitogen-activated protein kinase gene, VdHog1, regulates osmotic stress response, microsclerotia formation and virulence in *Verticillium dahliae*. Fungal Genet Biol 88:13–23. doi:10.1016/j.fgb.2016.01.011. 26812120

[14] Tian L, Wang Y, Yu J, Xiong D, Zhao H, Tian C. 2016. The mitogen-activated protein kinase kinase VdPbs2 of *Verticillium dahliae* regulates microsclerotia formation, stress response, and plant infection. Front Microbiol 7:1532. doi:10.3389/fmicb.2016.01532. 27729908 PMC5037172

[15] Yu J, Li T, Tian L, Tang C, Klosterman SJ, Tian C, Wang Y. 2019. Two *Verticillium dahliae* MAPKKKs, VdSsk2 and VdSte11, have distinct roles in pathogenicity, microsclerotial formation, and stress adaptation. MSphere 4:e00426-19. doi:10.1128/mSphere.00426-19. 31292234 PMC6620378

[16] Sarmiento-Villamil JL, Prieto P, Klosterman SJ, Garcia-Pedrajas MD. 2018. Characterization of two homeodomain transcription factors with critical but distinct roles in virulence in the vascular pathogen *Verticillium dahliae*. Mol Plant Pathol 19:986–1004. doi:10.1111/mpp.12584. 28727279 PMC6638091

[17] Tian L, Yu J, Wang Y, Tian C. 2017. The C_2_H_2_ transcription factor VdMsn2 controls hyphal growth, microsclerotia formation, and virulence of *Verticillium dahliae*. Fungal Biol 121:1001–1010. doi:10.1016/j.funbio.2017.08.005. 29122172

[18] Tang C, Jin X, Klosterman SJ, Wang Y. 2020. Convergent and distinctive functions of transcription factors VdYap1, VdAtf1, and VdSkn7 in the regulation of nitrosative stress resistance, microsclerotia formation, and virulence in *Verticillium dahliae*. Mol Plant Pathol 21:1451–1466. doi:10.1111/mpp.12988. 32954659 PMC7549003

[19] Almeida-Paes R, Frases S, Araújo G.dS, de Oliveira MME, Gerfen GJ, Nosanchuk JD, Zancopé-Oliveira RM. 2012. Biosynthesis and functions of a melanoid pigment produced by species of the *sporothrix* complex in the presence of _L_-tyrosine. Appl Environ Microbiol 78:8623–8630. doi:10.1128/AEM.02414-12. 23042177 PMC3502921

[20] Nosanchuk JD, Casadevall A. 2003. The contribution of melanin to microbial pathogenesis. Cell Microbiol 5:203–223. doi:10.1046/j.1462-5814.2003.00268.x. 12675679

[21] Liu GY, Nizet V. 2009. Color me bad: microbial pigments as virulence factors. Trends Microbiol 17:406–413. doi:10.1016/j.tim.2009.06.006. 19726196 PMC2743764

[22] Langfelder K, Streibel M, Jahn B, Haase G, Brakhage AA. 2003. Biosynthesis of fungal melanins and their importance for human pathogenic fungi. Fungal Genet Biol 38:143–158. doi:10.1016/s1087-1845(02)00526-1. 12620252

[23] Schumacher J. 2016. DHN melanin biosynthesis in the plant pathogenic fungus *Botrytis cinerea* is based on two developmentally regulated key enzyme (PKS)-encoding genes. Mol Microbiol 99:729–748. doi:10.1111/mmi.13262. 26514268

[24] Wheeler MH. 1982. Melanin biosynthesis in *Verticillium dahliae*: dehydration and reduction reactions in cell-free homogenates. Experimental MYCOLOGY 6:171–179. doi:10.1016/0147-5975(82)90091-3.

[25] Goncalves RC, Lisboa HC, Pombeiro-Sponchiado SR. 2012. Characterization of melanin pigment produced by *Aspergillus nidulans*. World J Microbiol Biotechnol 28:1467–1474. doi:10.1007/s11274-011-0948-3. 22805928

[26] Liu D, Wei L, Guo T, Tan W. 2014. Detection of DOPA-melanin in the dimorphic fungal pathogen *Penicillium marneffei* and its effect on macrophage phagocytosis in vitro. PLoS One 9:e92610. doi:10.1371/journal.pone.0092610. 24647795 PMC3960263

[27] Eisenman HC, Mues M, Weber SE, Frases S, Chaskes S, Gerfen G, Casadevall A. 2007. *Cryptococcus neoformans* laccase catalyses melanin synthesis from both _D_- and _L_-DOPA. Microbiology (Reading) 153:3954–3962. doi:10.1099/mic.0.2007/011049-0. 18048910

[28] Perez-Cuesta U, Aparicio-Fernandez L, Guruceaga X, Martin-Souto L, Abad-Diaz-de-Cerio A, Antoran A, Buldain I, Hernando FL, Ramirez-Garcia A, Rementeria A. 2020. Melanin and pyomelanin in *Aspergillus fumigatus*: from its genetics to host interaction. Int Microbiol 23:55–63. doi:10.1007/s10123-019-00078-0. 31020477

[29] Schmaler-Ripcke J, Sugareva V, Gebhardt P, Winkler R, Kniemeyer O, Heinekamp T, Brakhage AA. 2009. Production of pyomelanin, a second type of melanin, via the tyrosine degradation pathway in *Aspergillus fumigatus*. Appl Environ Microbiol 75:493–503. doi:10.1128/AEM.02077-08. 19028908 PMC2620705

[30] Kawamura C, Moriwaki J, Kimura N, Fujita Y, Fuji S, Hirano T, Koizumi S, T. 1997. The melanin biosynthesis genes of alternaria alternata can restore pathogenicity of the melanin-deficient mutants of *Magnaporthe grisea*. Mol Plant Microbe Interact 10:446–453. doi:10.1094/MPMI.1997.10.4.446. 9150594

[31] Frases S, Salazar A, Dadachova E, Casadevall A. 2007. *Cryptococcus neoformans* can utilize the bacterial melanin precursor homogentisic acid for fungal melanogenesis. Appl Environ Microbiol 73:615–621. doi:10.1128/AEM.01947-06. 17098915 PMC1796974

[32] Zhang T, Zhang B, Hua C, Meng P, Wang S, Chen Z, Du Y, Gao F, Huang J. 2017. *VdPKS1* is required for melanin formation and virulence in a cotton wilt pathogen *Verticillium dahliae*. Sci China Life Sci 60:868–879. doi:10.1007/s11427-017-9075-3. 28755294

[33] Yu D, Fang Y, Tang C, Klosterman SJ, Tian C, Wang Y. 2019. Genomewide transcriptome profiles reveal how Bacillus subtilis lipopeptides inhibit microsclerotia formation in Verticillium dahliae. Mol Plant Microbe Interact 32:622–634. doi:10.1094/MPMI-08-18-0233-R. 30489195

[34] Fang Y, Klosterman SJ, Tian C, Wang Y. 2019. Insights into VdCmr1-mediated protection against high temperature stress and UV irradiation in *Verticillium dahliae*. Environ Microbiol 21:2977–2996. doi:10.1111/1462-2920.14695. 31136051

[35] Nelson EB. 1990. Exudate molecules initiating fungal responses to seeds and roots. Plant Soil 129:61–73. doi:10.1007/BF00011692.

[36] Ali M, Cheng Z, Ahmad H, Hayat S. 2018. Reactive oxygen species (ROS) as defenses against a broad range of plant fungal infections and case study on ROS employed by crops against *Verticillium dahliae* wilts. J Plant Interactions 13:353–363. doi:10.1080/17429145.2018.1484188.

[37] Tang C, Xiong D, Fang Y, Tian C, Wang Y. 2017. The two-component response regulator VdSkn7 plays key roles in microsclerotial development, stress resistance and virulence of *Verticillium dahliae*. Fungal Genet Biol 108:26–35. doi:10.1016/j.fgb.2017.09.002. 28917999

[38] Griffiths DA. 1970. The fine structure of developing microsclerotia of *Verticillium dahliae* Kleb. Archiv Mikrobiol 74:207–212. doi:10.1007/BF00408881.

[39] Fan R, Klosterman SJ, Wang C, Subbarao KV, Xu X, Shang W, Hu X. 2017. *Vayg1* is required for microsclerotium formation and melanin production in *Verticillium dahliae*. Fungal Genet Biol 98:1–11. doi:10.1016/j.fgb.2016.11.003. 27866941

[40] Tian L, Xu J, Zhou L, Guo W. 2014. VdMsb regulates virulence and microsclerotia production in the fungal plant pathogen *Verticillium dahliae*. Gene 550:238–244. doi:10.1016/j.gene.2014.08.035. 25151308

[41] Pihet M, Vandeputte P, Tronchin G, Renier G, Saulnier P, Georgeault S, Mallet R, Chabasse D, Symoens F, Bouchara JP. 2009. Melanin is an essential component for the integrity of the cell wall of *Aspergillus fumigatus* conidia. BMC Microbiol 9:177. doi:10.1186/1471-2180-9-177. 19703288 PMC2740851

[42] Lehmann S, Serrano M, L'Haridon F, Tjamos SE, Metraux JP. 2015. Reactive oxygen species and plant resistance to fungal pathogens. Phytochemistry 112:54–62. doi:10.1016/j.phytochem.2014.08.027. 25264341

[43] Fernandes C, Mota M, Barros L, Dias MI, Ferreira I, Piedade AP, Casadevall A, Goncalves T. 2021. Pyomelanin synthesis in *Alternaria alternata* Inhibits DHN-melanin synthesis and decreases cell wall chitin content and thickness. Front Microbiol 12:691433. doi:10.3389/fmicb.2021.691433. 34512569 PMC8430343

[44] Boyce KJ, McLauchlan A, Schreider L, Andrianopoulos A. 2015. Intracellular growth is dependent on tyrosine catabolism in the dimorphic fungal pathogen *Penicillium marneffei*. PLoS Pathog 11:e1004790. doi:10.1371/journal.ppat.1004790. 25812137 PMC4374905

[45] Ketelboeter LM, M Ketelboeter L, Potharla VY, Y Potharla V, Bardy SL, L Bardy S. 2014. NTBC treatment of the Pyomelanogenic *Pseudomonas aeruginosa* clinical isolate PA1111 inhibits pigment production and increases sensitivity to oxidative stress. Curr Microbiol 69:343–348. doi:10.1007/s00284-014-0593-9. 24801336 PMC4113677

[46] Ketelboeter LM, Bardy SL. 2017. Characterization of 2–(2-nitro-4-trifluoromethylbenzoyl)-1,3-cyclohexanedione resistance in pyomelanogenic *Pseudomonas aeruginosa* DKN343. PLoS One 12:e0178084. doi:10.1371/journal.pone.0178084. 28570601 PMC5453437

[47] Del Sorbo G, Schoonbeek H, De Waard MA. 2000. Fungal transporters involved in efflux of natural toxic compounds and fungicides. Fungal Genet Biol 30:1–15. doi:10.1006/fgbi.2000.1206. 10955904

[48] Klein C, Kuchler K, Valachovic M. 2011. ABC proteins in yeast and fungal pathogens. Essays Biochem 50:101–119. doi:10.1042/bse0500101. 21967054

[49] Song TT, Zhao J, Ying SH, Feng MG. 2013. Differential contributions of five ABC transporters to multidrug resistance, antioxidion and virulence of *Beauveria bassiana*, an entomopathogenic fungus. PLoS One 8:e62179. doi:10.1371/journal.pone.0062179. 23596534 PMC3626590

[50] Klosterman SJ, Subbarao KV, Kang S, Veronese P, Gold SE, Thomma BPHJ, Chen Z, Henrissat B, Lee Y-H, Park J, Garcia-Pedrajas MD, Barbara DJ, Anchieta A, de Jonge R, Santhanam P, Maruthachalam K, Atallah Z, Amyotte SG, Paz Z, Inderbitzin P, Hayes RJ, Heiman DI, Young S, Zeng Q, Engels R, Galagan J, Cuomo CA, Dobinson KF, Ma L-J. 2011. Comparative genomics yields insights into niche adaptation of plant vascular wilt pathogens. PLoS Pathog 7:e1002137. doi:10.1371/journal.ppat.1002137. 21829347 PMC3145793

[51] Hawke MA, Lazarovits G. 1994. Production and manipulation of individual microsclerotia of *Verticillium dahliae* for use in studies of survival. PhytoPAthology (USA ) 84:883–890. doi:10.1094/Phyto-84-883.

[52] Goswami RS. 2012. Targeted gene replacement in fungi using a split-marker approach. Methods Mol Biol 835:255–269. doi:10.1007/978-1-61779-501-5_16. 22183659

[53] Larkin MA, Blackshields G, Brown NP, Chenna R, McGettigan PA, McWilliam H, Valentin F, Wallace IM, Wilm A, Lopez R, Thompson JD, Gibson TJ, Higgins DG. 2007. Clustal W and Clustal X version 2.0. Bioinformatics 23:2947–2948. doi:10.1093/bioinformatics/btm404. 17846036

[54] Tamura K, Stecher G, Peterson D, Filipski A, Kumar S. 2013. MEGA6: molecular evolutionary genetics analysis version 6.0. Mol Biol Evol 30:2725–2729. doi:10.1093/molbev/mst197. 24132122 PMC3840312

[55] Bournonville CF, Diaz-Ricci JC. 2011. Quantitative determination of superoxide in plant leaves using a modified NBT staining method. Phytochem Anal 22:268–271. doi:10.1002/pca.1275. 21360621

[56] Robinson MD, McCarthy DJ, Smyth GK. 2010. edgeR: a Bioconductor package for differential expression analysis of digital gene expression data. Bioinformatics 26:139–140. doi:10.1093/bioinformatics/btp616. 19910308 PMC2796818

[57] Chen C, Chen H, Zhang Y, Thomas HR, Frank MH, He Y, Xia R. 2020. TBtools: an integrative toolkit developed for interactive analyses of big biological data. Mol Plant 13:1194–1202. doi:10.1016/j.molp.2020.06.009. 32585190

