# Supplementary material for: The bZip Transcription Factor VdMRTF1 is a Negative Regulator of Melanin Biosynthesis and Virulence in Verticillium dahliae
Source: Microbiol Spectr. 2022 Apr 11;10(2):e02581-21. doi: 10.1128/spectrum.02581-21 (PMC9045294; doi:10.1128/spectrum.02581-21)
Supplement: SUPPLEMENTAL FILE 1 — Supplemental material. Download spectrum.02581-21-s0001.pdf, PDF file, 0.5 MB [file spectrum.02581-21-s0001.pdf]

Supplementary data

Figure S1. Phylogenetic analysis of VdMRTF1 of *Verticillium dahliae* and its homologs in other fungi.

Phylogenetic tree of *VdMRTF1* and its homologs in other fungi. All the amino acid sequences were aligned using the ClustalX program and the phylogenetic tree was viewed using a neighbor-joining method in MEGAX. The amino acid sequence of *VdMRTF1* and its homologs in following fungi were analyzed: *Verticillium dahliae* (VDAG_09790), *Verticillium longisporum* (BN1708_009494), *Colletotrichum truncatum* (CTRU02_03318), *Colletotrichum simmondsii* (CSIM01_00392), *Trichoderma gamsii* (TGAMA5MH_10608), *Fusarium graminum* (FGRMN_6821) and *Fusarium avenaceum* (FAVG1_12470). All proteins were downloaded from the NCBI database.

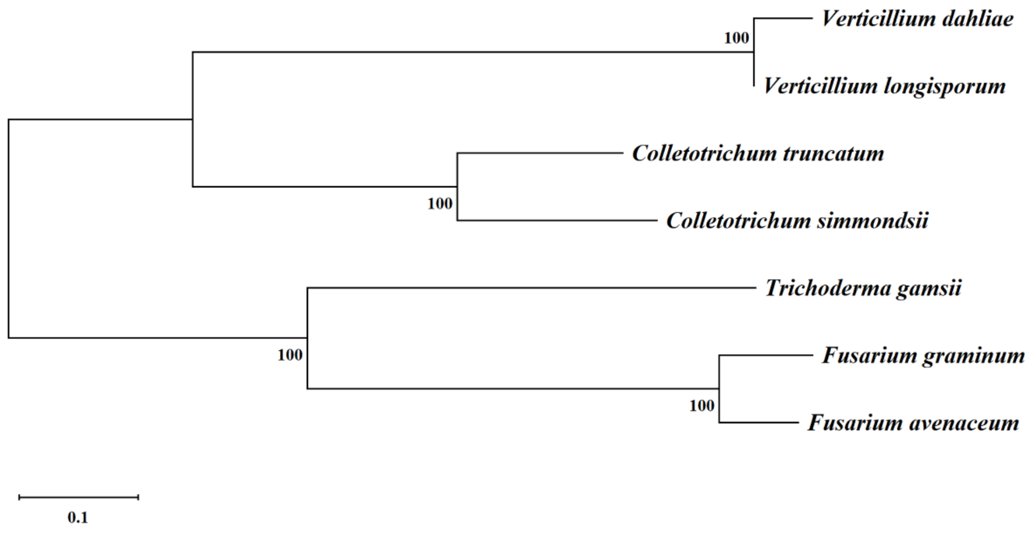

Figure S2. Verification of *ΔVdMRTF1* mutant in *V. dahliae.*

(A-C) The *VdMRTF1* knock-out and complementated strain were screened and confirmed by PCR and RT-PCR analysis. All primers are shown in Table1. **(D)** Confirmation of Δ*VdMRTF1* deletion mutants by Southern blotting. Genomic DNA of VdLs.17, Δ*VdMRTF1* and Δ*VdMRTF1*-C strains were digested with *Kpn*I. There is no band in the VdLs.17 strain, but a 3164 bp band in Δ*VdMRTF1* strain and Δ*VdMRTF1*-C strain.

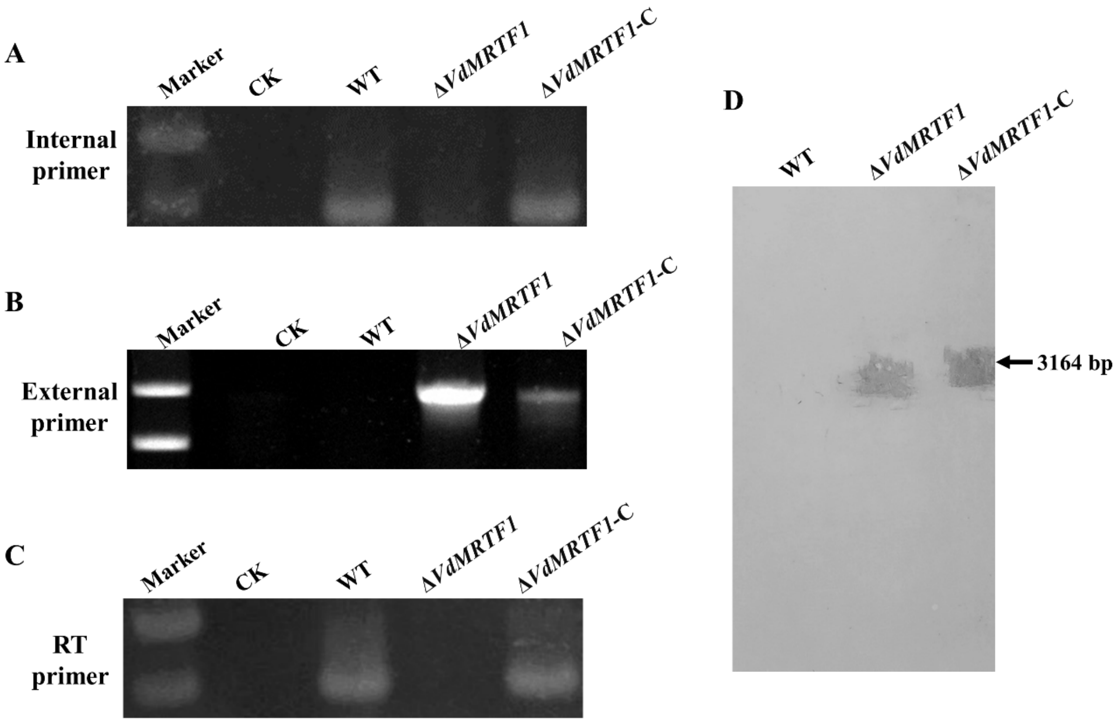

Figure S3. The differentially expressed genes of the wild type VdLs.17 and Δ*VdMRTF1* strains.

Bar chart indicating the number of DEGs (differentially expressed genes) in the wild type and Δ*VdMRTF1* strains.

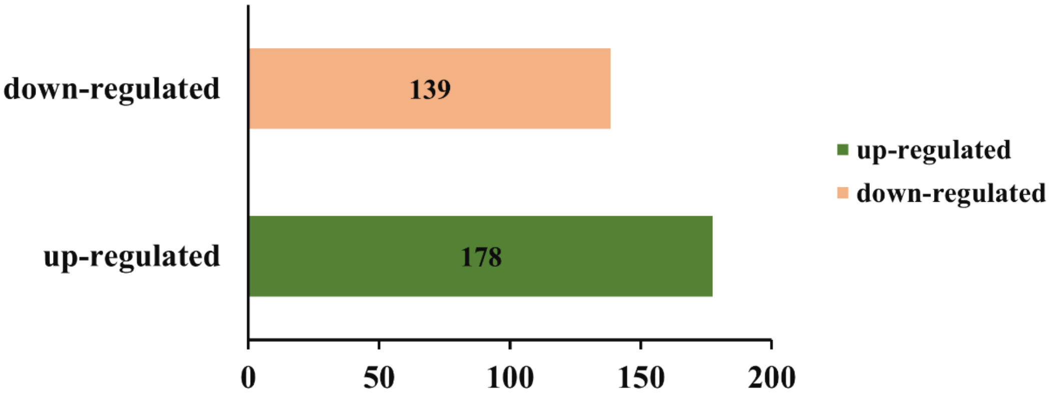

Figure S4. The Gene Ontology (GO) classification of differentially expressed genes between the wild type and *VdMRTF1* mutant strains in *V. dahliae.*

(A) Pie chart showing the GO classification of DEGs (differentially expressed genes) whose expression was increased after deletion of *VdMRTF1*. **(B)**The pie chart exhibiting GO classification of downregulated DEGs in Δ*VdMRTF1* strain. GO classification of DEGs was conducted according

to the GO resource (http://geneontology.org/).

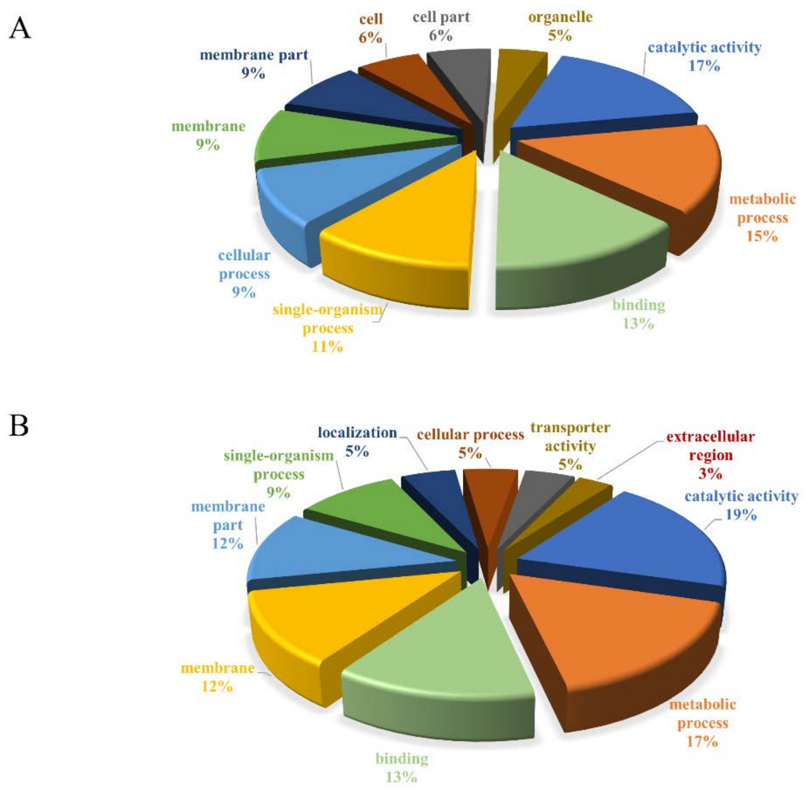

Figure S5. **The Kyoto Encyclopedia of Genes and Genomes (KEGG) classification and enrichment analyses of DEGs after loss of *VdMRTF1* in *V. dahliae.***

(A) The histogram illustrates the KEGG classification of genes differentially expressed between the wild type VdLs.17 and the Δ*VdMRTF1* strains. **(B)** A bubble chart indicates DEGs enriched in 20 KEGG pathways. The bubble size represents the number of DEGs. The X-axis represents the enrichment factor and the Y-axis represents KEGG pathway term.

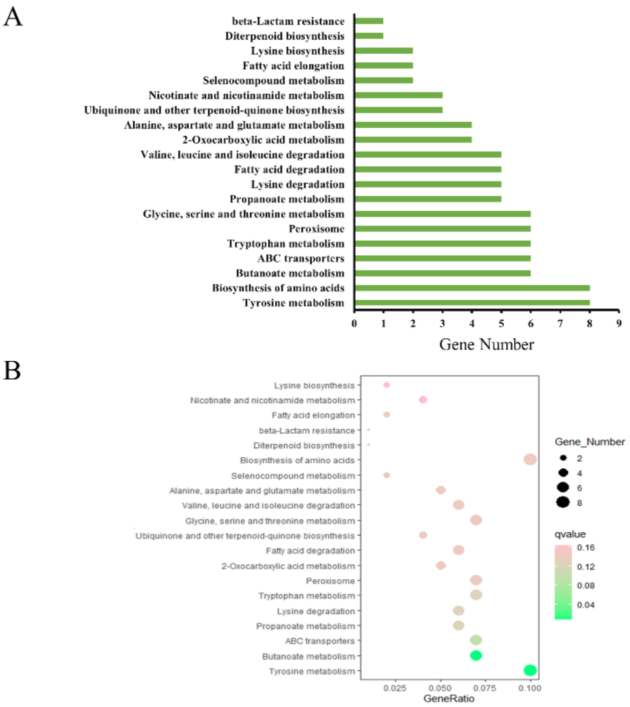

A
beta-Lactam resistance
Diterpenoid biosynthesis
Lysine biosynthesis
Fatty acid elongation
Selenocompound metabolism
Nicotinate and nicotinamide metabolism
Ubiquinone and other terpenoid-quinone biosynthesis
Alanine, aspartate and glutamate metabolism
2-Oxocarboxylic acid metabolism
Valine, leucine and isoleucine degradation
Fatty acid degradation
Lysine degradation
Propanoate metabolism
Glycine, serine and threonine metabolism
Peroxisome
Tryptophan metabolism
ABC transporters
Butanoate metabolism
Biosynthesis of amino acids
Tyrosine metabolism
0 1 2 3 4 5 6 7 8 9
Gene Number
B
Lysine biosynthesis
Nicotinate and nicotinamide metabolism
Fatty acid elongation
beta-Lactam resistance
Diterpenoid biosynthesis
Biosynthesis of amino acids
Selenocompound metabolism
Alanine, aspartate and glutamate metabolism
Valine, leucine and isoleucine degradation
Glycine, serine and threonine metabolism
Ubiquinone and other terpenoid-quinone biosynthesis
Fatty acid degradation
2-Oxocarboxylic acid metabolism
Peroxisome
Tryptophan metabolism
Lysine degradation
Propanoate metabolism
ABC transporters
Butanoate metabolism
Tyrosine metabolism
0.025 0.050 0.075 0.100
GeneRatio
Gene_Number
2
4
6
8
qvalue
0.16
0.12
0.08
0.04

Table S1. List of primers used in this study.

Primer name	Description	Primer sequence (5'-3')
bZIP25 5Fs<br>bZIP25 5Fx	5’ flanking sequence of VdMRTF1	TCCGTCACCAACCAGATA<br>GTTCTCCGACACTCTCCT
bZIP25 3Fs<br>bZIP25 3Fx	3’ flanking sequence of VdMRTF1	ACAGCAATCATGTGCATGG<br>GTCCCGCCTTATTCTGGT
bZIP25 ns<br>bZIP25 nx	Internal screening primer of VdMRTF1	CCAGCAATGAACAGACCTCG<br>TTGTAAAGCGGCGTACCTTG
bZIP25 zhs<br>bZIP25 zhx	External screening primer of VdMRTF1	CATGCATCAGACTTCCCAGC<br>CTCCATACAAGCCAACCACG
bZIP25 hbs<br>bZIP25 hbx	Primers for complementation of VdMRTF1	CCCGGACGTGACTACTTTTC<br>CCTTGTCCATGCACATGATT
P123<br>P124	Southern blot probe of VdMRTF1	CACCGCGACGTCTGTCGAGAAG<br>GGACGATTGCGTCGCATCGA
HYF<br>HYR	hygromycin- resistance cassette	CGTTGCAAGACCTGCCTGAA<br>GGATGCCTCCGCTCGAAGTA

Supplementary Table 2 Gene ontology annotation of the top 10 up-regulated and the top 10 down-regulated DEGs.

#ID	Regulated	GO_annotation
VDAG_07999	up	Molecular Function: kinase activity (GO:0016301)
VDAG_10373	up	--
VDAG_10085	up	Molecular Function: manganese ion transmembrane transporter activity (GO:0005384); Cellular Component: integral component of membrane (GO:0016021); Biological Process: cellular manganese ion homeostasis (GO:0030026)
VDAG_00321	up	--
VDAG_00047	up	Molecular Function: ATP binding (GO:0005524); Molecular Function: methyltransferase activity (GO:0008168)
VDAG_03345	up	Molecular Function: oxidoreductase activity, acting on the aldehyde or oxo group of donors, NAD or NADP as acceptor (GO:0016620)
NewGene_1021	up	--
VDAG_01167	up	Molecular Function: ATP binding (GO:0005524); Cellular Component: integral component of membrane (GO:0016021); Molecular Function: ATPase activity (GO:0016887); Molecular Function: ATPase activity, coupled to transmembrane movement of substances (GO:0042626)
VDAG_09115	up	Molecular Function: catalase activity (GO:0004096); Biological Process: response to oxidative stress (GO:0006979); Molecular Function: heme binding (GO:0020037); Biological Process: hydrogen peroxide catabolic process (GO:0042744); Molecular Function: metal ion binding (GO:0046872)
VDAG_00931	up	Biological Process: carbohydrate metabolic process (GO:0005975); Molecular Function: cellulase activity (GO:0008810)
VDAG_10412	down	--
VDAG_06734	down	Biological Process: carbohydrate metabolic process (GO:0005975); Molecular Function: chitin binding (GO:0008061)
VDAG_01547	down	--
VDAG_07711	down	Molecular Function: catalytic activity (GO:0003824); Cellular Component: integral component of membrane (GO:0016021)
VDAG_02280	down	--
VDAG_02655	down	--
VDAG_08070	down	--
VDAG_10100	down	Cellular Component: integral component of peroxisomal membrane (GO:0005779); Biological Process: peroxisome fission (GO:0016559)
VDAG_08214	down	Molecular Function: oxidoreductase activity (GO:0016491)
VDAG_02240	down	--
